# A Helix Replacement Mechanism Directs Metavinculin Functions

**DOI:** 10.1371/journal.pone.0010679

**Published:** 2010-05-19

**Authors:** Erumbi S. Rangarajan, Jun Hyuck Lee, S. D. Yogesha, Tina Izard

**Affiliations:** Cell Adhesion Laboratory, Department of Cancer Biology, The Scripps Research Institute, Scripps Florida, Jupiter, Florida, United States of America; University of Washington, United States of America

## Abstract

Cells require distinct adhesion complexes to form contacts with their neighbors or the extracellular matrix, and vinculin links these complexes to the actin cytoskeleton. Metavinculin, an isoform of vinculin that harbors a unique 68-residue insert in its tail domain, has distinct actin bundling and oligomerization properties and plays essential roles in muscle development and homeostasis. Moreover, patients with sporadic or familial mutations in the *metavinculin*-specific insert invariably develop fatal cardiomyopathies. Here we report the high resolution crystal structure of the metavinculin tail domain, as well as the crystal structures of full-length human native metavinculin (1,134 residues) and of the full-length cardiomyopathy-associated ΔLeu954 metavinculin deletion mutant. These structures reveal that an α-helix (H1′) and extended coil of the metavinculin insert replace α-helix H1 and its preceding extended coil found in the *N*-terminal region of the vinculin tail domain to form a new five-helix bundle tail domain. Further, biochemical analyses demonstrate that this helix replacement directs the distinct actin bundling and oligomerization properties of metavinculin. Finally, the cardiomyopathy associated ΔLeu954 and Arg975Trp metavinculin mutants reside on the replaced extended coil and the H1′ α-helix, respectively. Thus, a helix replacement mechanism directs metavinculin's unique functions.

## Introduction

The morphology and functions of specialized cells within tissues such as muscle requires unique organization of the actin cytoskeleton, but how this is controlled is poorly understood. At one level this relies on proper links of the actin network to cadherin receptor-mediated cell-cell adherens junctions, to integrin receptor-directed focal adhesions, and to intercalated discs that are required for muscle cell function and that orchestrate coordinated movement. These links are provided in part by vinculin, a highly conserved and structurally dynamic protein that stabilizes adhesions complexes [1] and which binds to actin through the agency of its five-helix bundle tail (Vt) domain [2]. Accordingly, in the mouse *vinculin* loss leads to defects in adhesion complexes that compromise embryonic cardiac development [3] and *vinculin^+/−^* mice develop dilated cardiomyopathy [4].

All muscle cell types selectively express an alternatively spliced isoform of vinculin, coined metavinculin, which harbors a 68-residue insert positioned between α-helices H1 and H2 of the Vt domain. Metavinculin levels are tightly controlled by signals regulating muscle function, where for example changes in mechanical load induce marked increases in levels of metavinculin protein [4], [5]. Importantly, metavinculin functions are also essential for maintaining the architecture of muscle actin-membrane attachment sites, as dilated cardiomyopathy (DCM) in man is associated with reductions in metavinculin levels [6]. Furthermore, recurrent familial and sporadic mutations in residues in the insert of *metavinculin*, like mutations in *actin*, are associated with severe idiopathic DCM [6], [7], [8]. Three such mutations in *metavinculin* have been described (Ala934Val, ΔLeu954, and Arg975Trp) and they differ in their severity of disease and effects on actin bundling. Specifically, the most severe mutant Arg975Trp is associated with both dilated (DCM) and hypertrophic (HCM) cardiomyopathies in man where it disrupts the organization of intercalated discs, results in a pI drop of about 1.4 pH units for residues 966–983, augments cross-linking of actin filaments [8], and may compromise the interactions of metavinculin with its partners, including vinculin [9]. By contrast, the ΔLeu954 and Ala934Val mutations are specifically associated with DCM and have more modest effects on the cross-linking of actin filaments, especially the Ala934Val mutation [7].

The functions of vinculin as a regulator of adhesion signaling and cell migration are well established [1], [10], [11], yet the specific functions that metavinculin plays in muscle cells remain obscure. Metavinculin is always co-expressed with vinculin in muscle but, unlike vinculin, it cannot homodimerize and rather forms heterodimers with vinculin, an interaction thought important for metavinculin functions [5]. Furthermore, metavinculin has been suggested to differ in the intramolecular interactions of its head and tail domains that clamp vinculin in its inactive, closed conformation [7], and it displays reduced binding to the acidic phospholipid PIP_2_, which promotes oligomerization of activated vinculin [5]. Importantly, although its insert does not bind to actin [5], metavinculin differs markedly from vinculin in its control of the actin network, where the metavinculin tail (MVt) domain (a surrogate for activated metavinculin) induces a fine meshwork of actin filaments while Vt provokes the formation of tight actin bundles [12]. Finally, MVt displays higher affinity for the hnRNP protein raver1 that co-localizes with metavinculin and vinculin at intercalated discs in cardiomyocytes [5], [13], [14], and which appears to deliver its mRNA cargo to nascent adhesion complexes via its interactions with activated vinculin [15], [16].

Vinculin is comprised of five helical bundle domains, four of which (Vh1, Vh2, Vh3, and Vt2) reside in the globular head (VH) of the molecule [17]. These are connected to Vt by a flexible proline-rich hinge domain that allows vinculin to spring open when the intramolecular contacts between its head and tail domains are severed by the binding of activators such as talin [18], [19], [20], [21]. By contrast, the structures of inactive and activated metavinculin are unknown.

Here, we present the first crystal structures of the human metavinculin tail domain and of full-length (124 kDa) metavinculin. These structures reveal that metavinculin harbors a unique α-helix (H1′) and a distinct preceding extended coil in its tail domain that replace the H1 α-helix and its preceding extended coil found in vinculin, which create a new five-helix bundle. Further, this helix replacement controls metavinculin's unique oligomerization and actin bundling functions. Finally, the crystal structure and analyses of metavinculin HCM and/or DCM mutants reveal that the unique architecture of metavinculin is affected by these fatal, recurrent mutations. Thus, a helix replacement mechanism mediates metavinculin functions.

## Results

### The crystal structures of metavinculin reveal a helix replacement mechanism

**Figure 1:**
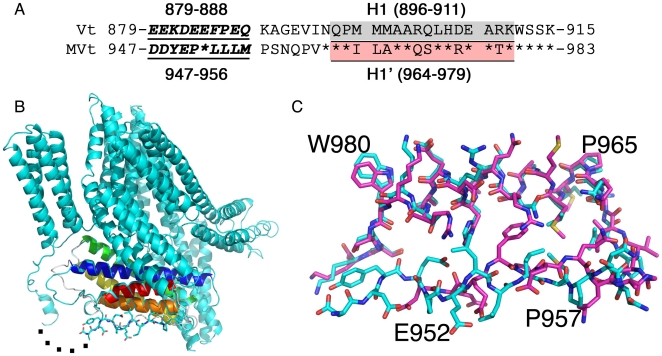
A Helix replacement mechanism is manifest in metavinculin. **A**: Structure based sequence alignment of α-helices H1 (underlined; grey background) and H1′ (underlined; pink background) and their preceding extended coil regions (underlined, bold, italicized). Residues 896–911 comprising α-helix H1 in human vinculin (Vt) show 56% identity to residues 964–979 comprising α-helix H1′ in metavinculin (MVt). Identical residues in MVt and Vt are indicated by an asterisk in the MVt sequence. **B**: Superposition of the metavinculin structure (residues 947–983; cyan) onto the vinculin tail (Vt) structure (residues 879–915; PDB entry 1rkE). The α-helices of Vt are colored in red (H1; residues 896–911), orange (H2; residues 918–937), yellow (H3; residues 944–972), green (H4; residues 975–1,005), and blue (H5; residues 1,014–1,046). An additional helix is seen in residues 850–854 in the proline-rich region of metavinculin, before the disordered region (residues 860–946), which is indicated by five dots. Some residues of metavinculin are labeled. The metavinculin extended coil is shown in ball-and-stick representation. **C**: Superposition of our full-length metavinculin structure (cyan) onto the vinculin tail structure (Vt, magenta; residues 879–1,066) showing the conformations of the distinct residues of the extended coil that precede α-helices H1 and H1′, respectively, in the two structures.

To define the molecular underpinnings of metavinculin's unique functions we solved the crystal structures of full-length human metavinculin (124 kDa) and its tail domain to 3.4 Å and 2.2 Å resolution, respectively (Figure 1; Tables 1 and 2). The structure of the globular head (VH) domain (residues 1–843) of metavinculin is nearly identical to that of vinculin [17] with a root mean square deviation (*r.m.s.d.*) of 1.57 Å for 575 C_α_ atoms when compared with that of vinculin.

**Table 1:** Data reduction statistics.

	Metavinculin	ΔLeu954	MVt-ΔH1
Space group	*P*4_3_2_1_2	*P*4_3_2_1_2	*R*3
Unit cell dimensions			
*a* = *b*	170.9 Å	171.1 Å	62.44 Å
*c*	210.9 Å	211.8 Å	62.44 Å
α = β = γ	90°	90°	111.87°
Resolution	50 Å–3.6 Å	50 Å–3.4 Å	50 Å–2.2 Å
Last shell	3.73 Å–3.6 Å	3.52 Å–3.4 Å	2.28 Å–2.2 Å
Total measurements	207,478	209,453	22,289
Unique reflections	36,379	42,424	11,090
Last shell	3,587	4,242	1,054
Wavelength	1 Å	0.97929 Å	0.97929 Å
R_sym_ a	0.083	0.053	0.087
Last shell	0.671	0.373	0.435
I/σ(I)	10.2	11.1	7.9
Last shell	2.9	4.3	1.9
Completeness	0.989	0.968	0.987
Last shell	0.994	0.986	0.947
Redundancy	5.7	5.0	2.1
Last shell	5.3	5.0	1.7

a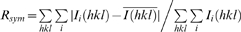.

**Table 2:** Crystallographic refinement statistics.

	Metavinculin	ΔLeu954	MVt-ΔH1
Resolution	50 Å–3.6 Å	50 Å–3.4 Å	50 Å–2.2 Å
Last shell	3.7 Å–3.6 Å	3.5 Å–3.4 Å	2.26 Å–2.2 Å
No. of reflections (working set)	34,508	40,136	10,411
No. of reflections (test set)	1,816	2,116	522
*R*_cryst_ a	0.226	0.237	0.198
Last shell	0.289	0.286	0.239
*R*_free_ b	0.255	0.269	0.258
Last shell	0.288	0.332	0.318
No. of amino acid residues	2,048	2,044	163
No. of solvent molecules			95
Average B-factor			
Protein	136.64 Å^2^	125.89 Å^2^	29.34 Å^2^
Solvent			39.43 Å^2^
*R.m.s.d.* from ideal geometry:			
Bond lengths	0.009 Å	0.009 Å	0.012 Å
Bond angles	1.09°	1.09°	1.195°

a
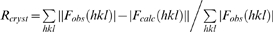

Where <|Fcalc|> is the expectation of |Fcalc| under the error model used in maximum-likelihood refinement.

b The free *R*-factor is a cross-validation residual calculated by using 5% of reflections which were randomly chosen and excluded from the refinement.

The 68-residue insert in metavinculin lies between α-helices H1 and H2 of Vt, between residues 915 and 916. It has been assumed that this domain would protrude from the 915–916 residue link, leaving the five-helix bundle of Vt unchanged. Surprisingly, our crystal structures show that this is not the case. Rather, an α-helix of the metavinculin insert (coined H1′) and its amino-terminal extended coil replace the H1 α-helix and its amino-terminal extended coil that are present in Vt, which are no longer part of the MVt domain but are rather disordered in the metavinculin structure (Figure 1; Supplementary Figures S1, S2, S3). Indeed, the H1′ for H1 replacement in metavinculin results in a new five-helix bundle (that is comprised of α-helices H1′ and H2–H5; residues 959–1,130) that closely mimics the five-helix bundle found in Vt (comprised of α-helices H1–H5; residues 891–1,062), where it can be superimposed with an *r.m.s.d.* of 0.69 Å (for 153 equivalent Cα positions). Thus, the true helical bundle MVt domain (residues 959–1,134) lacks the H1 α-helix of vinculin.

Recombinant four-helix H2–H5 protein was insoluble (ESR personal communication), which precluded a direct comparison of the binding of H1 versus H1′ to the H2–H5 helix bundle by conventional binding assays. However, a comparison of the tail domain structures of metavinculin with that of vinculin demonstrated that α-helix H1′ has several additional interactions with the four-helix bundle H2–H5 compared to α-helix H1 (Supplementary Figure S3). Specifically, there are two additional hydrophobic and hydrogen bond interactions in metavinculin, and there is also an electrostatic interaction of Arg-1107 with Glu-976 present in α-helix H1′, whereas the corresponding residues in Vt do not interact (Supplementary Figure S3). Further, the MVt five-helix bundle (α-helices H1′, H2–H5; residues 959–1130; MVt-ΔH1) has a significantly higher thermal melting temperature (72°C versus 67°C; Supplementary Figure S4) compared to the Vt five-helix bundle (α-helices H1, H2–H5; residues 891–1066; coined ΔN-Vt [lacking the extended amino-terminal extended coil of Vt]) consistent with the additional interactions directed by H1′ versus H1 seen in our crystal structures.

By contrast, the extended coil in metavinculin engages in fewer interactions with the metavinculin five-helix bundle compared to the distinct extended coil in Vt (Supplementary Figure S5, 6); indeed, this region is disordered in one metavinculin molecule in the asymmetric unit. The *N*-terminus of Vt affects binding to acidic phospholipids [22] and MVt is impaired in binding to acidic phospholipids [5]. The metavinculin structures now show that this region is distinct in the two vinculin isoforms, thus explaining their distinct PIP_2_ binding behaviors.

### Structures of metavinculin mutations associated with cardiomyopathies

**Figure 2:**
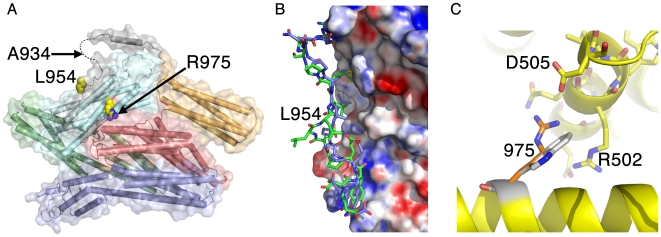
Crystal structure of the metavinculin-ΔLeu954 cardiomyopathy mutant. **A**: Native full-length metavinculin crystal structure showing the location of the three recurrent, autosomal dominant mutations, Ala934Val, ΔLeu-954, and Arg975Trp, that have been identified in familial and sporadic HCM and DCM. Arg975 and Leu954 are shown in space filling representation. **B**: Superposition of the native and metavinculin-ΔLeu954 crystal structure showing the differential extended coil formations (native, green; mutant, blue) and distinct interactions with the five-helix bundle tail domain. Electrostatic surface potential of <−20 to >+20 k_b_T, where k_b_ is the Boltzmann constant and T is the temperature (red, negative; blue, positive) is shown for the remainder of the tail domain which is close to identical for the two structures in the region shown. **C**: Modeling of the Arg975Trp mutation suggests that contacts found in the native metavinculin structure of the H1′ α-helix with the Vh3 seven-helix bundle (in particular eletrostatic interactions with Asp505) are lost in this severe HCM/DCM mutant.

Our full-length metavinculin crystal structure revealed that the severity of the autosomal dominant mutations in *metavinculin* identified in familial and sporadic HCM and DCM correlates with their location. The least severe Ala934Val substitution mutation resides on the displaced, disordered loop region that includes the proline-rich region, Leu-954 lies on the MVt extended coil, and the most severe mutation, Arg975Trp, resides on the unique H1′ α-helix of metavinculin (Figure 2A).

To define the effect of these HCM and DCM mutations on metavinculin structure, we determined the full-length crystal structure of the ΔLeu954 DCM deletion mutant (Figure 2B). Overall, the structure of full-length ΔLeu954-metavinculin resembles that of metavinculin, where superposition of the two full-length structures resulted in an *r.m.s.d.* of 0.54 Å for 1,031 Cα-atoms. Importantly, loss of Leu-954 results in a distinct conformation of the extended coil that precedes α-helix H1′ (Figure 2B) and this directs unique intramolecular interactions (Supplementary Figure S7).

The HCM/DCM mutant Arg975Trp has profound effects on the binding of metavinculin to actin [7], indicating that this mutation, which is positioned within α-helix H1′, should have substantial consequences on metavinculin structure. Indeed, recombinant full-length Arg975Trp metavinculin protein and its tail domain was prone to aggregation and degradation (ESR personal communication) precluding its structure determination. However, we modeled this mutation based on our native metavinculin structures (Figure 2C). In the metavinculin structure α-helix H1′ harboring Arg-975 is proximal to another helix (residues 492–505) from the Vh3 domain present in the globular head, where Arg-975 is involved in electrostatic interactions with Asp-505, which is evident in both molecules in the asymmetric unit. Additionally, the side chain of Arg-975 lies close to the side chain of Arg-502, allowing a possible hydrophobic stacking interaction in the Arg975Trp mutant. Notably, the electrostatic interaction of Arg-975 with Asp-505 is disabled by the introduction of the large hydrophobic side chain from tryptophan in the Arg975Trp HCM/DCM metavinculin mutant.

### Helix replacement does not affect the head∶tail interaction

The structure of full-length metavinculin also established that α-helix H1′ contributes to the VH-MVt interactions that clamp metavinculin in its inactive conformation [17], [22] (Supplementary Figure S3B, S3C). While residues that direct the interactions of the Vh1 head domain with MVt are identical to those used in the Vh1∶Vt interaction in vinculin, differences are found in the H1 versus H1′ interaction with the Vh3 domain of VH. Specifically, Arg-502 of the Vh3 domain interacts with Gln-904 on the vinculin α-helix H1 but with Ser-972 on the metavinculin α-helix H1′. Additionally, in metavinculin Arg-975 of α-helix H1′ interacts with Asp-505 of Vh3. Indeed, native gel analyses showed that MVt has a higher affinity for VH than that of the cardiomyopathy MVt-Arg975Trp mutant (Supplementary Figure S8A) in agreement with our modeling (Figure 2C). The effects were not evident in competition analyses with the cardiomyopahty-associated mutant MVt-ΔLeu954 (Supplementary Figure S8B), again in agreement with our structural analysis (Figure 2B). Nonetheless, the H1′ α-helix and its extended coil of metavinculin do not affect the overall interaction of native MVt for VH in solution, as MVt or Vt are equally capable of displacing each other from pre-existing complexes with VH (Supplementary Figure S8C).

### Metavinculin α-helix H1 is dispensable for actin binding

**Figure 3:**
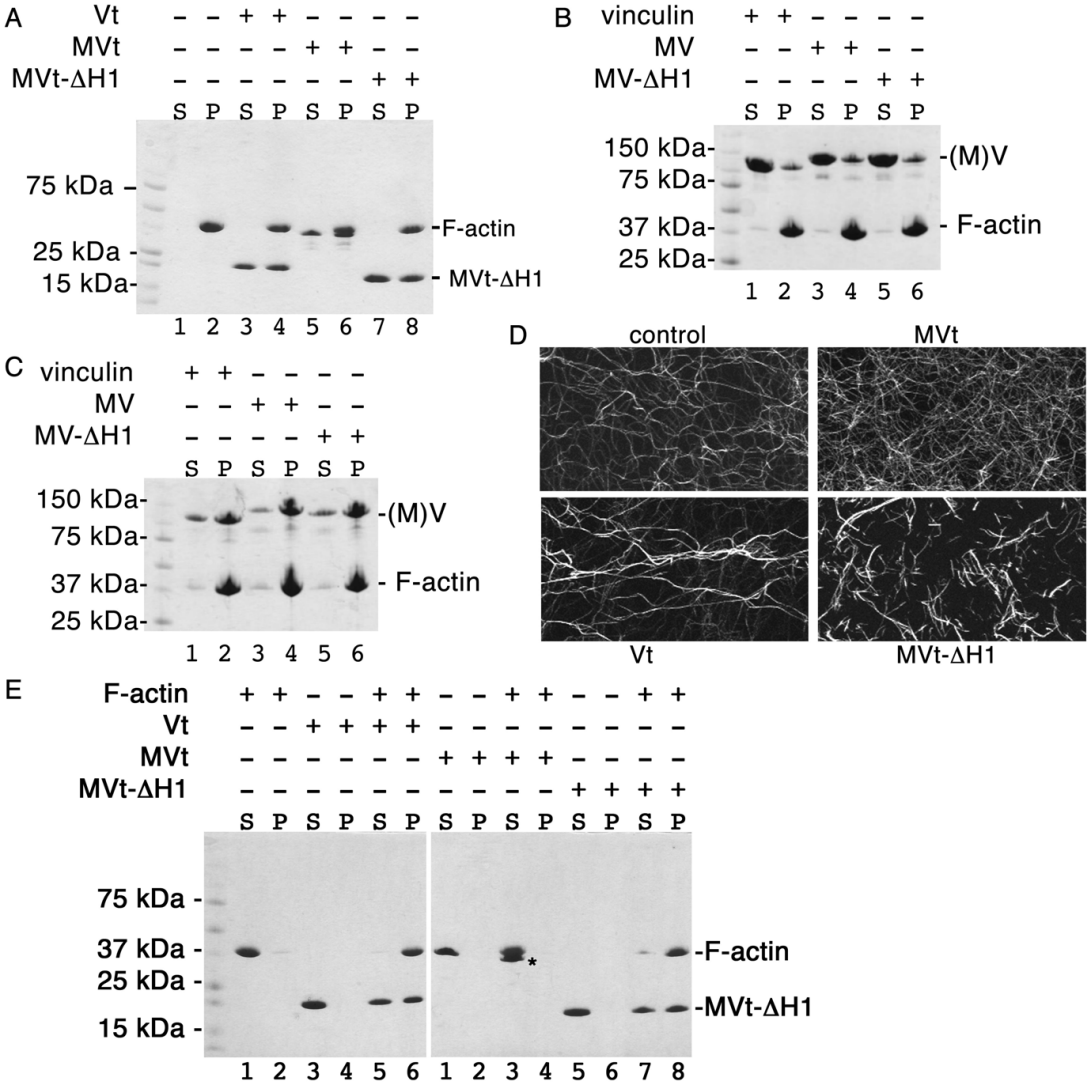
α-helix H1 directs the unique actin organizing properties of metavinculin. **A**: Vt (lanes 3, 4), MVt (lanes 5, 6), or MVt-ΔH1 (lanes 7, 8) or full-length **B**: inactive or **C**: talin-VBS3 activated vinculin (lanes 1, 2), metavinculin (lanes 3, 4), or MV-ΔH1 (lanes 5, 6) were tested for their ability to co-sediment F-actin (S, supernatant; P, pellet). Note that following activation by binding with talin-VBS3 (**C**), there is a substantial increase in the proportion of F-actin that is bound by vinculin, metavinculin and metavinculin-ΔH1 (**B**), and that there is no difference in binding to F-actin for MV-ΔH1 compared to metavinculin or vinculin. Therefore, α-helix H1 is dispensable for metavinculin binding to F-actin, which is functionally and structurally replaced by metavinculin α-helix H1′. The three vinculin proteins (vinculin, metavinculin, and MV-ΔH1) are indicated as ‘(M)V’. **D**: Fluorescence microscopy analyses of TRITC-phalloidin labeled F-actin alone, or when incubated with 0.2 µM MVt, Vt, or MVt-ΔH1. Note that loss of α-helix H1 from MVt converts this into a vinculin-like molecule that provokes the formation of tight actin bundles, whereas MVt provokes a meshwork of actin filaments. We obtained similar results with 0.4 µM tail domains (data not shown). The width of each individual panel corresponds to about 80 µm. **E**: F-actin bundling efficiency of Vt (left gel; lanes 5, 6), MVt (right gel; lanes 3, 4; asterisk) and MVt-ΔH1 (right gel; lanes 7, 8) were analyzed by low speed centrifugation (S, supernatant; P, pellet). Vt on the left gel, which migrates slightly less than MVt-ΔH1, is not labeled. Control reactions containing only F-actin (left gel; lanes 1, 2), Vt (left gel; lanes 3, 4), MVt (right gel; lanes 1, 2), or MVt-ΔH1 (right gel; lanes 5, 6) were also run to show that the proteins by themselves do not pellet under the experimental conditions. Representative experiment carried out in duplicate is shown.

Vinculin activation is required for binding to F-actin, which is mediated by the Vt domain [23]. The MVt structure, where α-helix H1′ replaces H1 to mimic Vt, suggested that α-helix H1 was dispensable for actin binding by metavinculin. Indeed, actin co-sedimentation assays demonstrated that MVt-ΔH1 and metavinculin-ΔH1 can bind to F-actin (Figure 3A, 3C) and that metavinculin-ΔH1 binding was, as expected, greatly augmented by talin-VBS3 (Figure 3B, 3C), a known activator of vinculin [24]. Thus, the novel five-helix bundle tail domain of metavinculin indeed directs actin binding.

### Metavinculin α-helix H1 directs differential actin organizing functions

To address whether the disordered α-helix H1 played important roles in the unique actin bundling properties of metavinculin we assessed the effects of deleting α-helix H1 on actin filament organization. Fluorescence microscopy of TRITC-phalloidin labeled F-actin demonstrated that, as expected [7], [12], Vt induced actin bundles whereas MVt induced a meshwork of actin filaments (Figure 3D). Notably, deletion of α-helix H1 from MVt converted this into a vinculin-like molecule that induced actin bundles (Figure 3D). Similar effects were seen in low speed actin bundling experiments where MVt-ΔH1 displays similar bundling effect as Vt (Figure 3E). Thus, the displaced H1 α-helix directs the differential actin organizing functions of metavinculin.

### The H1′ α-helix of metavinculin impairs homodimerization

**Figure 4:**
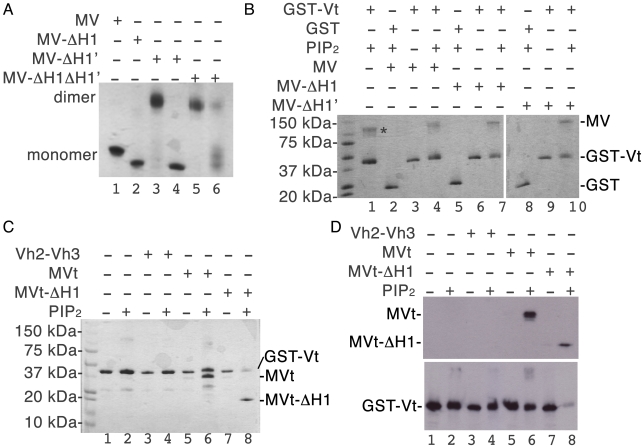
α-Helix H1′ impairs metavinculin homodimerization but not heterodimerization with vinculin. **A**: α-helix H1′ impairs metavinculin homodimerization. Native gel analyses of full-length metavinculin (MV, lane 1), metavinculin-ΔH1 (lane 2), metavinculin-ΔH1′ (lanes 3 and 4), and metavinculin-ΔH1ΔH1′ (lanes 5 and 6) are shown. Metavinculin and metavinculin-ΔH1 are monomers in solution, while metavinculin-ΔH1′ and metavinculin-ΔH1ΔH1′ both form monomers and dimers as determined by sizing chromatography (Supplementary Figure S9). Peak fractions for each species were run on this native gel (lanes 3–6). The identities of the two oligomeric species of metavinculin-ΔH1′ and metavinculin-ΔH1ΔH1′ that were separated by gel filtration chromatography (lanes 3–6) were confirmed by western blot analyses (data not shown). **B**: Vinculin∶metavinculin interactions as analyzed by pull-down assay and SDS PAGE gel analyses. Purified GST-Vt was tested for its ability to bind to full-length metavinculin (MV, lanes 2–4), metavinculin-ΔH1 (lanes 5–7), or metavinculin-ΔH1′ (lanes 8–10) +/− PIP_2_. Protein identity was confirmed by Western blotting (data not shown): the band marked with an asterisk is a GST-Vt homodimer, which was also observed all other lanes where GST-Vt and PIP_2_ is present (lanes 4, 7, and 10). No non-specific binding was observed of metavinculin to GST (lanes 2, 5, and 8). **C**: SDS-PAGE analysis of GST-Vt interactions with MVt. Purified GST-Vt was tested for its ability to bind to MVt and Vh2–Vh3 (negative control) +/− PIP_2_. As with the full-length proteins (**B**), heterodimerization is observed of GST-Vt with MVt (lane 6) and MVt-ΔH1 (lane 8) but not with the Vh2–Vh3 domains (lanes 3, 4). **D**: The His-tag Western blot (top panel; 1 min exposure time) shows no non-specific binding to Vh2–Vh3 but strong binding to MVt (lane 6) and MVt-ΔH1 (lane 8) in the presence of PIP_2_. The GST Western blot (bottom panel; 3 min exposure time) shows GST-Vt presence in all lanes.

Vinculin oligomerization effectively amplifies its interactions with other binding partners to stabilize adhesion junctions [25], [26]. Vt readily dimerizes and trimerizes in the presence of the acidic phospholipid phosphatidylinositol-4,5-bisphosphate (PIP_2_), whereas MVt fails to form homodimers but does heterodimerize with Vt [5]. Our metavinculin structures suggested that the H1′ α-helix and its preceding extended coil might impair oligomerization of metavinculin. Indeed, full-length metavinculin lacking that H1′ α-helix (metavinculin-ΔH1′), as well as metavinculin lacking both H1′ and H1 α-helices (metavinculin-ΔH1ΔH1′) readily form homodimers in solution independent of PIP_2_ (Figure 4A; Supplementary Figure S9). By contrast, metavinculin and metavinculin-ΔH1 are only found as monomers. Interestingly, deletion of either or both of these α-helices did not affect heterodimerization with Vt (Figure 4B–D). Therefore, the H1′ α-helix impairs metavinculin homodimerization but does not affect metavinculin∶vinculin interactions.

## Discussion

The studies presented herein establish that an α-helix and extended coil replacement mechanism controls metavinculin structure, oligomerization, and F-actin bundling functions. The helix replacement is directed by the unique intramolecular interactions of the H1′ α-helix and its preceding extended coil of the metavinculin insert with the H2–H5 helical bundle present in the tail domain, which replaces the H1 α-helix and its preceding extended coil that are present in the five-helix bundle tail domain of vinculin. Notably, this event explains many of the properties ascribed to metavinculin, where we have shown that the replacement of the H1 α-helix controls metavinculin's unique actin organizing properties and directs metavinculin oligomerization. Finally, the importance of helix replacement is underscored by the findings that recurrent and severe *metavinculin* mutations found in cardiomyopathies are positioned within or immediately preceding to the H1′ α-helix and that the Arg975Trp mutation affects the head∶tail interaction.

**Figure 5:**
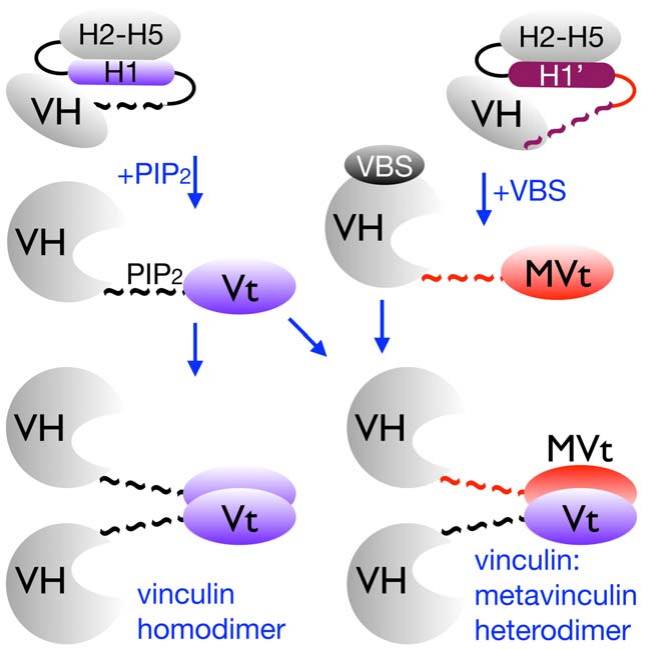
Helix and extended coil replacement control metavinculin oligomerization. Binding of PIP_2_ is thought to promote vinculin oligomerization through tail∶tail interactions [25]. Metavinculin on the other hand does not bind to PIP_2_ nor homodimerize [5] and our binding studies (Figure 4) show that metavinculin interacts with Vt∶PIP_2_. Further, our metavinculin crystal structures (Figure 1) show that the *N*-terminus of Vt thought to be involved in PIP_2_ binding [22] is distinct in metavinculin, thus explaining why metavinculin does not bind to PIP_2_. Finally, metavinculin heterodimerization does not require α-helices H1 or H1′ (Figure 4) suggesting that heterodimerization occurs via the metavinculin four-helix (helices H2–H5) bundle. The vinculin head domain VH is shown in gray, the distinct extended coils are represented by “∼ ∼” (in black or magenta for Vt or MVt, respectively) while the remainder of Vt or MVt is shown in blue or red, respectively. For clarity, the disordered α-helix H1 of metavinculin is not shown.

The H1′ α-helix restricts metavinculin oligomerization, as deletion of this helix triggers the spontaneous formation of metavinculin homodimers. Thus, one level of control of metavinculin-vinculin heterodimerization with vinculin as seen in muscle cells may involve the acidic phospholipid PIP_2_ and vinculin, as PIP_2_ is thought to promote vinculin oligomerization by unfurling the tail domain of activated vinculin [2], whereas metavinculin has a reduced affinity for PIP_2_ relative to vinculin [5]. Although what region of vinculin's tail domain binds to PIP_2_ is still not resolved [2], [22], [27], [28], [29], [30], PIP_2_ does bind to Vt variants that perturb interactions between the extended coil with the five-helix bundle more readily than to intact Vt [22]. This suggests that regions that are distinct in the two crystal structures of MVt and Vt harbor the PIP_2_ binding site, which may explain the impaired binding of PIP_2_ to MVt [22]. Collectively, we posit that PIP_2_ binding may promote metavinculin∶vinculin heterodimer formation by unfurling Vt [2] and allowing the Vt interaction with MVt (Figure 5). A portion of MVt can likely unfurl without PIP_2_ in solution, as the extended coil that precedes H1′ is disordered in one molecule of the metavinculin crystal structure. However, further unfurling of MVt might be blocked by its distinct α-helix H1′, which we posit impairs PIP_2_ binding and which appears to have a higher affinity for the H2–H5 four-helix bundle than the α-helix H1 in vinculin. Such a scenario would explain why metavinculin does not self-oligomerize and why its interactions are restricted to those with vinculin (Figure 5).

Muscle tissue homeostasis requires proper levels and function of metavinculin, a fact underscored by the clinical association between DCM and reduced levels of metavinculin [9] as well as by the recurrent mutations in the *metavinculin* insert in HCM and DCM [7], [9]. Of the three recurrent mutations Arg975Trp is located on the H1′ α-helix, and helix replacement is necessary to impart the unique actin bundling properties of metavinculin, which requires the replaced H1 α-helix (Figure 3C). Similarly, Leu-954 resides on the extended coil that precedes H1′ in metavinculin. The Arg975Trp mutation introduces a large hydrophobic patch in H1′ α-helix, whereas loss of Leu-954 provokes a distinct conformation of the extended coil. These findings establish that critical structural features of this subdomain orchestrate the actin network in a fashion required for muscle cell homeostasis.

## Materials and Methods

### Analysis of melting temperature by circular dichroism (CD), Head-tail displacement assays, Dynamic light scattering (DLS) analysis

Details regarding CD, displacement assays, and DLS experiments are all provided in the Supplementary Methods (Methods S1).

### Expression constructs for metavinculin and vinculin proteins

Human *metavinculin* cDNA (gi|50593530) was generated by PCR cloning the additional 68 residue (204 bp) insert, using long oligonucleotides and our *vinculin* cDNA construct [17], and was cloned into the pET3 expression vector (Novagen). The final expression construct was a *C*-terminal octa-histidine (His_8_) fusion tag and included all residues (1–1,134) of human metavinculin.

For biochemical studies, full-length human vinculin, metavinculin, and deletion mutants of metavinculin (metavinculin-ΔH1, lacking residues 895–915; metavinculin-ΔH1′, lacking residues 963–983; and metavinculin-ΔH1ΔH1′, lacking residues 895–915 and 963–983) were cloned into a modified pET28 vector using *NdeI* and *NotI* cloning sites to obtain a precision protease cleavable *N*-terminal His_8_-tagged fusion constructs. For metavinculin-ΔH1, the *N*-terminal (residues 1–894) and *C*-terminal (residues 916–1,134) sequences were first amplified independently and the resultant PCR products were subsequently used as a template for amplification of the entire stretch using the *N*-terminal forward primer and the *C*-terminal reverse primer to obtain metavinculin-ΔH1 amplicons. A similar approach was used to clone metavinculin-ΔH1′, in which the *N*-terminal (residues 1–962) and *C*-terminal (residues 984–1,134) segments were first amplified and then used as templates to obtain metavinculin-ΔH1′ amplicons. Metavinculin-ΔH1 was used as a template to obtain metavinculin-ΔH1ΔH1′ clones. All clones were transformed into BL21-DE3 (Novagen) for expression of the His_8_-tagged fusion proteins.

The full-length DCM metavinculin mutant ΔLeu954 was cloned using traditional molecular biology techniques.

MVt-ΔH1 (residues 959–1,130) was cloned into a modified pET28 vector using the *Nde*I and *BamH*I cloning sites, to obtain an *N*-terminal His_8_-tagged fusion protein having a precision protease cleavage site. The GST tagged vinculin tail domain (GST-Vt; residues 879–1,066) was obtained by cloning Vt in pGEX-4T-1 (GE Healthcare) using the *BamHI* and *EcoRI* cloning sites. ΔN-Vt (residues 891–1,066) was cloned into pGEX-6P-1 (GE Healthcare) using *BamHI* and *NotI* cloning sites. In addition, Vt (residues 879–1,066), MVt (residues 879–1,134), MVt-ΔLeu954 (residues 879–1133), and MVt-R975W (residues 879–1134) were also cloned in pET28, using *Nde*I and *Xho*I cloning sites, to obtain cleavable *N*-terminal hexa-histidine tagged fusion constructs.

The head domain of vinculin (VH; residues 1–843) was cloned into a modified pET3 vector using *NcoI* and *NotI* restriction sites to obtain a precision protease cleavable *C*-terminal His_8_-tagged fusion protein. The truncated head domain (Vh2–Vh3; residues 260–718) was cloned into a modified pET28 vector using *NdeI* and *NotI* cloning sites to produce a precision protease cleavable *N*-terminal His_8_-tagged fusion protein. The head domain containing α-helix H1 of vinculin (VH-H1; residues 1–915) was cloned into the pET-28 vector using *NdeI* and *SalI* restriction sites toΔ obtain a thrombin cleavable *N*-terminal His_6_ tag. All constructs were verified by sequencing.

### Protein expression and purification

Expression of recombinant proteins (His_8_-tagged full-length human metavinculin, metavinculin-ΔLeu954, VH, Vh2–Vh3, MVt, MVt-ΔLeu954, MVt-R975W, Vt, GST-Vt, and GST-ΔN-Vt) was performed by growing transformed BL21-DE3 cells at 37°C in Luria-Bertani medium containing appropriate antibiotics. Protein expression was induced at OD_600_ = 0.8 with 0.5 mM IPTG and cells were harvested after incubation at 30°C for 24 hr.

Pelleted cells of His-tagged full-length human metavinculin, re-suspended in 20 mM Tris-HCl (pH 7) and 150 mM NaCl, was lysed in presence of EDTA-free protease inhibitor cocktail (Roche) by passing through French Pressure Cell (2 cycles) and clarified by ultracentrifugation (100,000×*g* for 45 min). The cell lysate was loaded on a HisTrap chelating nickel affinity column (GE Healthcare) and washed extensively with 20 mM Tris-HCl and 150 mM NaCl (38 column volumes). The bound protein was then eluted over a gradient of 0.5 M imidazole (pH 8) and pooled peak fractions were dialyzed extensively against 25 mM CAPS (pH 10) buffer. The dialyzed protein sample was further purified using ion-exchange chromatography, by applying the sample on Hi-Trap QHP column (GE Healthcare) pre-equilibrated in 25 mM CAPS (pH 10) buffer. The bound protein was eluted using a salt gradient of up to 1 M NaCl in 25 mM CAPS (pH 10) and, following concentration, the protein was loaded onto a preparative Superdex-200 26/60 column (GE Healthcare) pre-equilibrated in 20 mM Tris (pH 8) and 150 mM NaCl. Purified metavinculin protein was then buffer exchanged to 10 mM Tris (pH 8) and 1 mM DTT and concentrated to 22 mg/ml for crystallization.

Similar expression and purification protocols were employed for purifying the metavinculin-ΔLeu954 and the His_8_-tagged truncated mutants metavinculin-ΔH1, metavinculin-ΔH1′, and metavinculin-ΔH1ΔH1′. The purified protein of metavinculin-ΔLeu954 was concentrated to 22 mg/ml in the final buffer containing 10 mM Tris (pH 8) and 1mM DTT and the metavinculin-ΔH1, metavinculin-ΔH1′, and metavinculin-ΔH1ΔH1′ proteins were concentrated to 9.5, 6.8, and 2.6 mg/ml respectively, in 20 mM Tris (pH 8) and 150 mM NaCl.

For the production of selenomethionine (SeMet)-labeled full-length metavinculin, the metavinculin expression plasmid was transformed into B834 methionine auxotroph cells and grown overnight at 37°C in Luria-Bertani medium (containing 20 mg/l ampicillin). The cells were separated by centrifugation at 3,000×*g* for 20 min, washed twice with sterile M9 minimal media and inoculated into pre-formulated SeMet media (Molecular Dimensions Ltd.) and incubated for 4 hr at 37°C. Protein production was induced with 0.5 M IPTG and cells were harvested after 24 hr incubation at 30°C. The purification of SeMet-labeled metavinculin was similar to wild type full-length metavinculin. The protein was concentrated to 22 mg/ml in 10 mM Tris (pH 8) and 1 mM DTT and used for crystallization.

Pelleted cells (VH, VH-H1, MVt, MVt-ΔLeu954, MVt-R975W, or Vt) were re-suspended and lysed by sonication in 100 ml of 50 mM NaH_2_PO_4_ (pH 8), 300 mM NaCl, and 5 mM imidazole. The lysate was then clarified by ultracentrifugation (100,000×*g* for 45 min) and the supernatant applied to HisTrap chelating nickel affinity column (GE Healthcare) and washed extensively with sonication buffer (22 column volumes). The bound protein was then eluted over a gradient of 1 M imidazole (pH 8). Affinity tags were removed by overnight cleavage with thrombin (VH-H1, MVt, MVt-ΔLeu954, MVt-R975W, or Vt) or precision protease (MVt-ΔLeu954 and MVt-R975W) at 4°C, and all proteins were further purified by gel filtration using a Superdex-200 (VH or VH-H1) or Superdex-75 (MVt, MVt-ΔLeu954, MVt-R975W, or Vt) column (GE Healthcare) pre-equilibrated in 20 mM Tris-HCl (pH 8) and 150 mM NaCl.

MVt-ΔH1 was produced using 500 ml of auto-induction media grown at 30°C for 20 hr in presence of 40 mg/l of kanamycin [31]. The cell pellet was re-suspended in 20 mM Tris-HCl, 200 mM NaCl and 5 mM Imidazole (pH 8) and lysed by sonication. Following clarification by ultracentrifugation (100,000×*g* for 45 min), the cell lysate was applied on HisTrap chelating nickel affinity column (GE Healthcare). After extensive washing (20 column volumes) in the lysis buffer, bound His-tagged MVt-ΔH1 was eluted over a gradient of 0.5 M imidazole (pH 8) in 20 mM Tris-HCl (pH 8) and 200 mM NaCl. Following elution, the His_8_ tag was removed by overnight treatment with precision protease at 4°C and the concentrated MVt-ΔH1 sample was further purified using a Superdex-75 column (GE Healthcare), pre-equilibrated with 20 mM Tris (pH 8) and 400 mM NaCl. The purified protein was concentrated to 11 mg/ml in the final buffer of 20 mM Tris-HCl (pH 8) and 400 mM NaCl.

Vh2–Vh3 was purified similar to MVt-ΔH1, however the His_8_ tag was not removed and the size exclusion chromatography on a Superdex-75 column was carried out using 20 mM Tris-HCl (pH 8) and 150 mM NaCl. The purified protein (6.6 mg/ml) was used for biochemical studies.

For GST-Vt purification, the cell pellet was re-suspended in 20 mM Tris-HCl (pH 8) and 150 mM NaCl, lysed by sonication and clarified by ultracentrifugation (100,000×*g* for 45 min). Subsequently, the supernatant was applied onto GSTrap FF column and after extensive washing with the buffer (20 mM Tris-HCl (pH 8) and 150 mM NaCl) the bound GST-Vt was eluted with 20 mM reduced glutathione (pH 8). The eluted protein was further purified on a 26/60 Superdex 75 column that was pre-equilibrated with 20 mM Tris-HCl (pH 8) and 150 mM NaCl and was used for all biochemical studies at a concentration of 1.8 mg/ml.

ΔN-Vt was purified by affinity chromatography on GST-Sepharose similar to GST-Vt. Following elution, GST-ΔN-Vt was incubated with precision protease overnight at 4°C in the presence of 20 mM Tris-HCl (pH 8), 400 mM NaCl, 1 mM DTT, and 1 mM EDTA. After concentration, the cleaved protein was purified on a 26/60 Superdex 75 column (GE Healthcare), pre-equilibrated with 20 mM Tris-HCl (pH 8), 400 mM NaCl, 1 mM DTT, and 1 mM EDTA to obtain purified ΔN-Vt. The protein was concentrated to 9.7 mg/ml for CD experiments.

### Crystallization and X-ray data collection

The Hauptman-Woodward High Throughput Crystallization Facility identified several similarly shaped full-length human metavinculin crystals from comparable conditions. After extensive optimization, these crystals grew up to 0.4 mm^3^ over a period of several weeks. Despite significant efforts to improve these metavinculin crystals, diffraction was highly mosaic, to at best 8 Å Bragg spacings. Systematic screening of hundreds of crystals at the Advanced Photon Source (APS) at Argonne National Laboratory (ANL) beamlines (SBC-CAT ID and SER-CAT) resulted in one complete native data set to 4.2 Å resolution. SDS-PAGE gel analysis of these crystals showed that some degradation occurred during crystal growth over several weeks at room temperature (data not shown). Therefore, we harvested crystals within a few days, as soon as their size reached 0.1 mm^3^–0.2 mm^3^. A few of these smaller sized crystals, produced by hanging drop vapor diffusion using a reservoir solution of 0.6 M Na/K phosphate and 175 mM ammonium sulfate, diffracted X-rays to 3.6 Å Bragg spacings at APS-ANL with much improved spot shape. The native metavinculin crystallization conditions were also used for the SeMet metavinculin and metavinculin-ΔLeu954 and the latter crystals were streak seeded to obtain a 3.4 Å data set at the APS-ANL SER-CAT 22ID beam line. Native and mutant metavinculin crystals belong to the tetrahedral space group *P*4_3_2_1_2, with unit cell dimensions *a* = *b* = 171.5 Å and *c* = 211.4 Å and two molecules in the asymmetric unit (Z = 16), a volume to mass ratio, V_M_ [32], of 3.13 Å^3^/Da, and a solvent content of about 60%. The data collection statistics are provided in Table 1.

All images were integrated, scaled, and merged using HKL2000 [33]. SeMet-metavinculin data sets were also collected to 4.4 Å Bragg spacings at the peak of the Se K absorption spectrum. Full-length human metavinculin crystals were extremely sensitive to radiation, especially at the Se peak wavelength. An attenuation of 75% (corresponding to 25% transmission) and 5 seconds exposure time allowed complete data set collection from a single crystal.

MVt-ΔH1 crystals were obtained by sitting drop vapor diffusion from 2.5 M NaCl and 100 mM sodium acetate (pH 4.5). Crystals of size less than 50 microns were obtained within 36 hr and appeared as needle clusters. A single crystal was dissected from the cluster, flash frozen directly in liquid nitrogen after a brief transfer into a cryo-protection solution containing reservoir solution supplemented with 35% (v/v) glycerol. Crystals of MVt-ΔH1 belong to rhombohedral space group *R*3, with unit cell dimensions *a* = *b* = *c* = 62.4 Å and α = β = γ = 111.9° and one molecule in the asymmetric unit (Z = 9) with a volume to mass ratio, V_M_ [32], of 2.9 Å^3^/Da corresponding to a solvent content of about 57%. X-ray diffraction data were collected at the APS SBC-CAT 19ID beam line and processed using HKL3000 [34]. The data collection statistics are provided in Table 1.

### Structure determination and crystallographic refinement

We obtained the initial phases for metavinculin from our original 4.2 Å native data set by molecular replacement using full-length vinculin (PDB entry 1tr2) [17] as the search model in the program MOLREP [35]. However, crystallographic refinement was stalled due to model bias at this resolution. Experimental phases obtained from a 4.4 Å SeMet metavinculin data set allowed us to overcome over fitting. Of the 76 Se in the asymmetric unit, 60 were identified by anomalous difference Fourier calculation and were further validated using the program SHARP [36]. The resulting experimental map was used to model the *C*-terminal tail domain of metavinculin and Se sites were used to discern the *N*-terminal helix identity of the tail domain. The Se sites allowed us to rule in or out possible rearrangements of the helical bundles. Indeed, Vt has three methionines in its H1 α-helix and none in its *N*-terminal strand, while MVt has one methionine in its *N*-terminal strand but none it in its H1′ α-helix, which is positioned in the H1 binding site of Vt. Thus, the experimental phases provided an unbiased map that suggested that instead of an insertion of a domain at residue 915, the metavinculin insert replaces vinculin α-helix H1 with α-helix H1′ from the insert. Crystallographic refinement was carried out using the 3.6 Å native data set. After several rounds of model building into both molecules in the asymmetric unit, the NCS operators were refined sufficiently to provide an average map that showed a high level of side chain detail and, together with the Se positions and our high resolution MVt-ΔH1 structure, allowed unambiguous tracing. Low resolution as well as variable domain movement between the two subunits in the asymmetric unit were limiting factors in applying non-crystallographic symmetry based refinement using REFMAC [35]. Thus, autoBUSTER was used in the final rounds of refinement, after each cycle of manual model building, where non-crystallography symmetry restraints were applied through implementing local structure similarity restrains (LSSR) [37], [38] along with application of group B-factor for individual residues. Omit maps, calculated using SFCHECK [39], and B-factor sharpened maps were used to achieve better tracing of the main chain as well as some of the side chains. However, at this resolution disordered regions were difficult to parameterize in terms of multiple conformations, which limited the scope of reliably assigning bound water molecules. Remarkable improvement in the model quality was achieved by refinement with autoBUSTER through implementation of local structure similarity restraints (LSSR) [38] and manual building using Coot [40]. The refinement statistics for metavinculin are provided in Table 2.

The full-length metavinculin structure was used as a search model to solve the structure of metavinculin-ΔLeu954 by molecular replacement using the program MOLREP [35]. Rigid body refinement was followed by restrained refinement with REFMAC5 [35]. Subsequently, all refinement was carried out using autoBUSTER by specific implementation of LSSR [38] and manual model building was achieved with Coot [40]. The refinement statistics for metavinculin-ΔLeu954 are provided in Table 2.

The final structures of metavinculin and metavinculin-ΔLeu954 contained residues 1–834, 954–1,114 and 1,122–1,130 in subunit A and residues 1–859 and 947–1,130 in subunit B. Due to lack of electron density and disordered nature, the residues 835–953, 1,115–1,121, 1,131–1,134 in subunit A and residues 860–946 and 1,131–1,134 in subunit B were not included in the final model.

The structure of MVt-ΔH1 was solved by molecular replacement using the Vt domain of Vh∶Vt complex (PDB entry 1rke) [19] as the search model in the program MOLREP [35]. Rigid body refinement was followed by iteration of restrained refinement with REFMAC5 [35] and manual model building into 2*F_obs_-F_calc_* maps using Coot [40]. The final refined model comprises residues 960–1,116 and 1,122–1,130. Due to poor electron density, the region encompassing residues 959 and 1,117–1,121 were not included in the final model. The refinement statistics for MVt-ΔH1 are provided in Table 2.

### Actin binding and bundling analyses

Actin pull-down assays were carried out as described [24] either in PBS or in 20 mM Tris-HCl (pH 8) and 150 mM NaCl. Briefly, 30 µM of freshly polymerized F-actin was incubated at room temperature for 20 min with 60 µM each of the tail domains of vinculin (Vt) and metavinculin (MVt and MVt-ΔH1). Subsequently, after ultracentrifugation at 95,000×g at 25°C for 15 min, the supernatant and the pellet were analyzed on SDS-polyacrylamide gels and the protein bands visualized by staining with Coomassie Blue. Similarly, actin binding experiments were carried out for inactive and active full-length vinculin (15 µM), metavinculin (14 µM), or MV-ΔH1 (15 µM) with F-actin (30 µM). Activation of full-length vinculin or metavinculin was achieved by incubating the proteins with 20-fold molar excess of talin-VBS3 peptide at room temperature for 10 min prior to mixing with F-actin.

Actin bundling analysis using fluorescence microscopy was performed using F-actin labeled with TRITC-phalloidin as described [41]. Skeletal muscle actin (Cytoskeleton) in G-actin buffer (0.2 M Tris-HCl pH 8, 0.2 mM ATP, 0.5 mM DTT, and 0.2 mM CaCl_2_) was allowed to polymerize at a concentration of 1 mg/ml in F-actin buffer (1 mM imidazole pH 7.4, 1 mM ATP, 0.1 M KCl, and 2 mM MgCl_2_) for 1 hr at 37°C. Labeling of F-actin was performed by incubating 500 µl of F-actin buffer with 20 µl of polymerized F-actin (0.8 µM final actin concentration) and 10 µl of TRITC-phalloidin (0.1 mg/ml stock in DMSO) for 2 hr on ice. The effect of various vinculin and metavinculin tail domains on F-actin was investigated by mixing half volumes (0.5∶1) of the target proteins (0.2 µM final concentration) with TRITC-phalloidin labeled F-actin (0.4 µM final actin concentration) and incubating at 37°C for 1 hr. Aliquots of the mixture were then placed on a parafilm and covered with poly-L-lysine coated glass cover slip (Fisher Scientific) and incubated further for 1 hr at 4°C. The adsorbed protein on the cover slip was fixed with 3.7% formaldehyde for 20 min and mounted after washing thrice with PBS. The fluorescent images of F-actin were recorded with an Olympus FluoView 1,000 Confocal Microscope and processed using FV10-ASW software (Olympus).

All F-actin bundling analyses using low speed centrifugation was carried out using freshly polymerized F-actin. Briefly, actin (purchased from Cytoskeleton Inc, CO, USA) was polymerized in F-actin buffer (20 mM Tris-HCl pH 8, 10 mM MgCl_2_, 10 mM ATP, 0.2 mM CaCl_2_ and 0.1 M KCl) at 5 mg/ml for 30 min at room temperature and precisely after the incubation time the F-actin solution is further diluted with equal volume of PBS to obtain F-actin at a final concentration of 2.5 mg/ml. F-actin solution was then spun at 7,500 *r.p.m.* for 10 min and the supernatant is used immediately for sedimentation assays. The above procedure for preparation of F-actin seems to be critical as any deviation tend to produce more F-actin in the pellets including that of the control experiment. Bundling analyses were carried out using 30 µM F-actin (optimal concentration required to see bundling effect) with equimolar concentration of the tail domains. The tail domains (Vt, MVt or MVt-ΔH1) were incubated with or without F-actin at room temperature for 10 min and centrifuged at low speed (6,000 *r.p.m.*; Eppendroff table top centrifuge) for 10 min. The supernatant was removed without disturbing the pellet and stored separately while the pellet was washed gently by layering with 100 µl of PBS and removing the excess buffer. The last step was repeated gently three more times without disturbing the pellet and subsequently, the supernatant and pellet were analyzed on SDS-polyacrylamide PHAST gels and protein bands visualized with Coomassie blue staining.

### GST pull-down dimerization analyses

One ml of purified GST-Vt (0.5 µM; 20 mM Tris-HCl, 150 mM NaCl) was incubated with 40 µl of GST-Sepharose beads on a rotary shaker at room temperature for 30 min. Beads were then washed three times with 1 ml buffer containing 20 mM Tris-HCl pH 8 and 150 mM NaCl to eliminate any trace amounts of unbound GST-Vt. In one set of experiments, bound GST-Vt was then further incubated with 10-fold molar excess (5 µM) of phosphatidylinositol-4,5-bisphoshpate (PIP_2_) at 37°C for 1 hr. PIP_2_ solution (choloroform∶methanol∶water ratios of 20∶9∶1) as obtained from the manufacturer (Avanti Polar Lipids, Inc.) was vacuum dried and re-suspended in 20 mM Tris-HCl (pH 8) and 150 mM NaCl to a final concentration of 5 mg/ml. Prior to the experiment, the PIP_2_ solution was incubated at 42°C for 2 hr and diluted to the final concentration in the reaction mixture. The final concentration of PIP_2_ (5 µM) used in the reaction was well below the measured CMC value [42]. Following incubation beads were subjected to extensive washing (three cycles of 1 ml each) with the buffer to remove excess PIP_2_. In the second set of experiments, GST-Vt was used directly for the binding experiment to compare the binding efficiency of metavinculin in the absence of PIP_2_. Subsequently, 15 µM of “pre-activated” (using talin-VBS3) His_8_-tagged metavinculin or metavinculin deletion mutants were incubated with either PIP_2_-treated or untreated GST-Vt for 1 hr at 37°C. The beads were then washed three times with 1 ml buffer and GST-Vt bound metavinculin heterodimer was eluted in 20 µl of 20 mM Tris-HCl and 150 mM NaCl containing 20 mM reduced glutathione and analyzed by SDS-PAGE. Similarly, formation of GST-Vt heterodimer with MVt was also analyzed using a truncated vinculin head domain (Vh2–Vh3) as a negative control. Proteins were identified by Western blot analysis using HRP-conjugated αHis or αGST antibodies.

## Supporting Information

Methods S1 Supplementary Methods.
(0.03 MB DOC)

Figure S1 Helix replacement mechanism in metavinculin. Structure-based sequence alignment of the α-helices of vinculin (Vt) and metavinculin (MVt) tail domains aligns the Vt α-helix H1 with the MVt α-helix H1′ and their respective *N*-terminal extended coils. This was a surprising revelation from the crystal structure since it was assumed that the α-helix H1 that is identical in sequence in the two isoforms would also be structurally conserved. The α-helices of MVt are underlined and colored in grey (H1′), red (H1), orange (H2), yellow (H3), green (H4), and blue (H5). The structurally equivalent extended coil regions are in bold, underlined, and italicized. The Vt extended coil region (residues 879–888) is underlined in the MVt sequence. Identical residues in MVt and Vt are indicated by an asterisk in the Vt sequence.
(3.44 MB TIF)

Figure S2 MVt electron density map. Stereo view of the final 2*F_obs_-F_calc_* electron density map at 2.2 Å of the metavinculin tail domain contoured at 1σ around the replaced α-helix H1′ represented in sticks. The remaining four α-helices are shown as a cartoon. The α-helices of MVt are colored in orange (H2; residues 986–1,004), yellow (H3; residues 1,012–1,038), green (H4; residues 1,043–1,071), and blue (H5; residues 1,081–1,114) while the α-helix H1′ (residues 964–979) is shown in sticks.
(2.52 MB TIF)

Figure S3 Intramolecular interactions of α-helices H1 in vinculin versus H1′ in metavinculin. Schematic of the intramolecular interactions of α-helix H1 of Vt (A) versus those of α-helix H1′ of MVt (B). The residues binding to Vt α-helix H1 (boxed in pale blue) or MVt α-helix H1′ (boxed in peach) are shown on the left (A) or on the right (B) of the respective helices. Residues are distinguished according to the type of their interaction (hydrophobic, white; hydrogen bonds, gray; backbone hydrogen bonds, blue; electrostatic interactions, pink). The asterisks indicate additional interactions found in MVt (Ser-1002, Arg-1006, Arg-1107) or altered interactions compared to Vt (Ser-972 with Lys-1103 in MVt versus Gln-904 with Lys-1035 in Vt). C: Cartoon stereo drawing of the full-length metavinculin crystal structure. The head domain, VH, is shown in pink (Vh1 sub-domain; residues 1–258) and gray (residues 259–840) and the tail domain, MVt, is shown in blue (residues 946–963 and 980–1,132) and yellow (α-Helix H1′; residues 964–979). α-Helix H1′ is shown in yellow and is not involved in the Vh1∶MVt interaction. The vinculin and metavinculin structure, including the distinct α-helices H1 and H1′, superimpose well (as shown in the superposition depicted in Figure 1) and, accordingly, α-helix H1 in vinculin is also not involved in the Vh1∶Vt interface. The entire head domain (residues 1–843) of metavinculin shows a root mean square deviation (*r.m.s.d.*) of 1.57 Å for 575 Cα atoms when compared with that of vinculin, while the MVt domain (residues 946–1,132) exhibits an *r.m.s.d.* of 0.8 Å for 163 Cα atoms. The termini as well as the disordered region are labeled (“N” and “C”, and “855” and “946”, respectively).
(6.93 MB TIF)

Figure S4 MVt is more stable than Vt. Normalized thermal unfolding curves of ΔN-Vt (residues 891–1066; red) and MVt-ΔH1 (residues 959–1130; blue) monitored at 222 nm are shown. The T_m_ values indicate that the metavinculin five-helix bundle (72°C) is significantly more stable compared to the vinculin five-helix bundle (67°C).
(1.34 MB TIF)

Figure S5 The extended coil in metavinculin engages in fewer interactions than that of the extended coil of vinculin. Stereo view of the Vt (A) and MVt (B) domains of the full-length metavinculin and vinculin crystal structures reveals that the extended coil in vinculin engages in more intradomain interactions compared to metavinculin.
(6.92 MB TIF)

Figure S6 Fewer intramolcular interactions of the extended coil of metavinculin compared to the extended coil of vinculin. Schematic of the intramolecular interactions of the extended coil of vinculin (A) versus those of metavinculin (B). A: Six extended coil residues (three of these in only one subunit, indicated by the red and blue lines) interact with thirteen Vt residues, including five electrostatic interactions. B: Five metavinculin-specific extended coil residues engage in eleven hydrophobic/hydrogen bonding interactions. Indeed, these interactions are only seen in one subunit (indicated by the blue line), as the metavinculin-specific extended coil is disordered in the other subunit.
(2.13 MB TIF)

Figure S7 Intramolecular interactions of the extended coil in metavinculin and in the Leu954 deletion metavinculin mutant associated with cardiomyopathies. Residues binding to the wild type extended coil (A; boxed in light blue) or mutant metavinculin (B; boxed in peach) are shown on the left (A) or right (B) of the respective coils. Residues are distinguished according to the type of their interaction (hydrophobic, white; hydrogen bonds, gray). The deletion of Leu-954 is indicated by a dotted line in panel B. Due to the deletion, the numbering for residues 955–1,134 of wild type metavinculin in the ΔL954 mutant is 954–1,133.
(2.17 MB TIF)

Figure S8 Helix replacement does not affect the head∶tail interaction. Reciprocal tail displacement native gel analyses of VH (residues 1–843) in complex with (A) MVt-Arg975Trp, (B) MVt-ΔLeu954, and (C) Vt versus MVt (A and C) or MVt-ΔH1(B). VH in complex with MVt or MVt-ΔLeu954 migrate similarly (ESR personal communication); thus, MVt-ΔH1 was used instead of MVt to be able to distinguish the complexes. Vt and MVt-ΔH1 do not migrate into the gel due to their basic pIs of 9.32 and 9.89, respectively. Competing tail domains were titrated (arrows) into preformed complexes at 2- and 10-fold molar excess. Notably, the DCM-associated metavinculin mutant MVt-R975W (A) shows the weakest head∶tail interaction while the tail domains of the two vinculin isoforms or the ΔLeu deletion mutant do not affect this interaction. Representative analyses of native gels are shown. The less-than sign (<) and asterisk highlight the displaced VH∶tail domain complexes. MVt displaces MVt-Arg975Trp more readily (A), MVt-ΔLeu954 and MVt-ΔH1 displace each other similarly (B), and MVt and Vt displace each other equally (C).
(2.17 MB TIF)

Figure S9 α-Helix H1′ impairs metavinculin oligomerization. A: Chromatogram of size exclusion chromatography of full length metavinculin (red; 123.8 kDa), MV-ΔH1 (green; 121.3 kDa), and MV-ΔH1′ (blue; 121 kDa) on a Hiload 26/60 Superdex 200 column calibrated with standard molecular weight markers (thyroglobulin, 669 kDa; ferritin, 440 kDa; aldolase, 158 kDa; conalbumin, 75 kDa; and ovalbumin, 43 kDa). The elution positions of the standard proteins are indicated on the chromatogram. The shaded areas for the dimer (‘d’) and monomer (‘m’) elutants of MV-ΔH1′ indicate the fractions pooled for dynamic light scattering (panels B and C) and native gel (Figure 4A) experiments. B: and C: Analysis of the oligomeric state of the two species, ‘d’ and ‘m’, of MV-ΔH1′ separated by gel filtration (A) by dynamic light scattering (DLS) at 23°C. DLS measurements were carried out at a scattering angle of 90° on a Dynapro Titan (Wyatt technologies) dynamic light scattering apparatus. D: The calculated molecular weight (MWR) for peak fractions of the two species of MV-ΔH1 separated by gel filtration (A) as determined by dynamic light scattering. Values provided correspond to major peaks as obtained by DLS experiment. The relative intensity in this panel represents the relative amount of light scattered by each population of species in solution while the relative intensities in panels B and C indicate the relative amount of light scattered by the bin compared to the other bin and thus differ in values for the same peak.
(2.60 MB TIF)
