# Supplementary material for: A Helix Replacement Mechanism Directs Metavinculin Functions
Source: PLoS One. 2010 May 19;5(5):e10679. doi: 10.1371/journal.pone.0010679 (PMC2873289; doi:10.1371/journal.pone.0010679)
Supplement: Methods S1 — Supplementary Methods. (0.03 MB DOC) [file pone.0010679.s001.doc]

**Supplementary Methods**

**Analysis of melting temperature by Circular Dichroism (CD)**

CD measurements for untagged ΔN-Vt and MVt-ΔH1 were carried out on a Jasco J-815 spectropolarimeter (JASCO Inc, Maryland, USA) equipped with a Peltier single position thermostatic cell holder controlled by a Jasco Peltier temperature control system. Since ΔN-Vt was stable in 20 mM Tris  (pH 8), 400 mM NaCl, 1 mM EDTA, and 1 mM DTT, all measurements were carried out in the same buffer at a concentration of 0.2 mg/ml and 1 mm path length. Initial spectra were recorded at 22°C in far-UV range between 200 nm to 260 nm to obtain the wavelength of maximum signal change, which was recorded as 222 nm. To measure the melting temperature (T_m_), thermal unfolding was carried out by monitoring the signal change at 222 nm over a temperature range of 40°C - 90°C at an interval of 1°C and a temperature slope of 1°C/min. Spectra Manager software provided with the CD instrument was used for data collection and analysis.

**Head-tail displacement assays**All binding analyses were performed in 20 mM Tris-HCl (pH 8) and 150 mM NaCl. Initially, the complexes of various tail domains of vinculin (Vt) and metavinculin (MVt, MVt-ΔH1, and MVt-ΔL954) with the head domain VH were pre-formed by mixing equimolar concentrations (10 μM) and incubating for 10 min at room temperature. Complete VH:MVt-R975W complex formation was achieved using 1:2 molar ratio. Following incubation, 2- and 10-fold molar excess of the competing tail domain was added and incubated for additional 10 min and analyzed by non-denaturating polyacrylamide PHAST gels and visualized using Coomassie blue staining.

**Dynamic light scattering analysis**

MV, MV-ΔH1, and MV-ΔH1’ peak samples obtained by size exclusion chromatography were analyzed by dynamic light scattering on a Dynapro-Titan (Wyatt Technology Corporation) instrument at a protein concentration of 2 mg/ml in 20 mM Tris-HCl (pH 8) and 150 mM NaCl. All protein samples were spun at maximum speed (14,000 r.p.m.) on a table top centrifuge for 20 min prior to taking measurements at ambient temperature and scattering angle of 90°. Typical experiments consisted of 10 acquisitions of 10 sec each and the estimated distribution of the particles size based on the measured hydrodynamic radius and subsequent calculation of molecular mass was carried out using the DYNAMICS version 6.0 software.
