# Supplementary figures and images for: A Helix Replacement Mechanism Directs Metavinculin Functions
Source: PLoS One. 2010 May 19;5(5):e10679. doi: 10.1371/journal.pone.0010679 (PMC2873289; doi:10.1371/journal.pone.0010679)

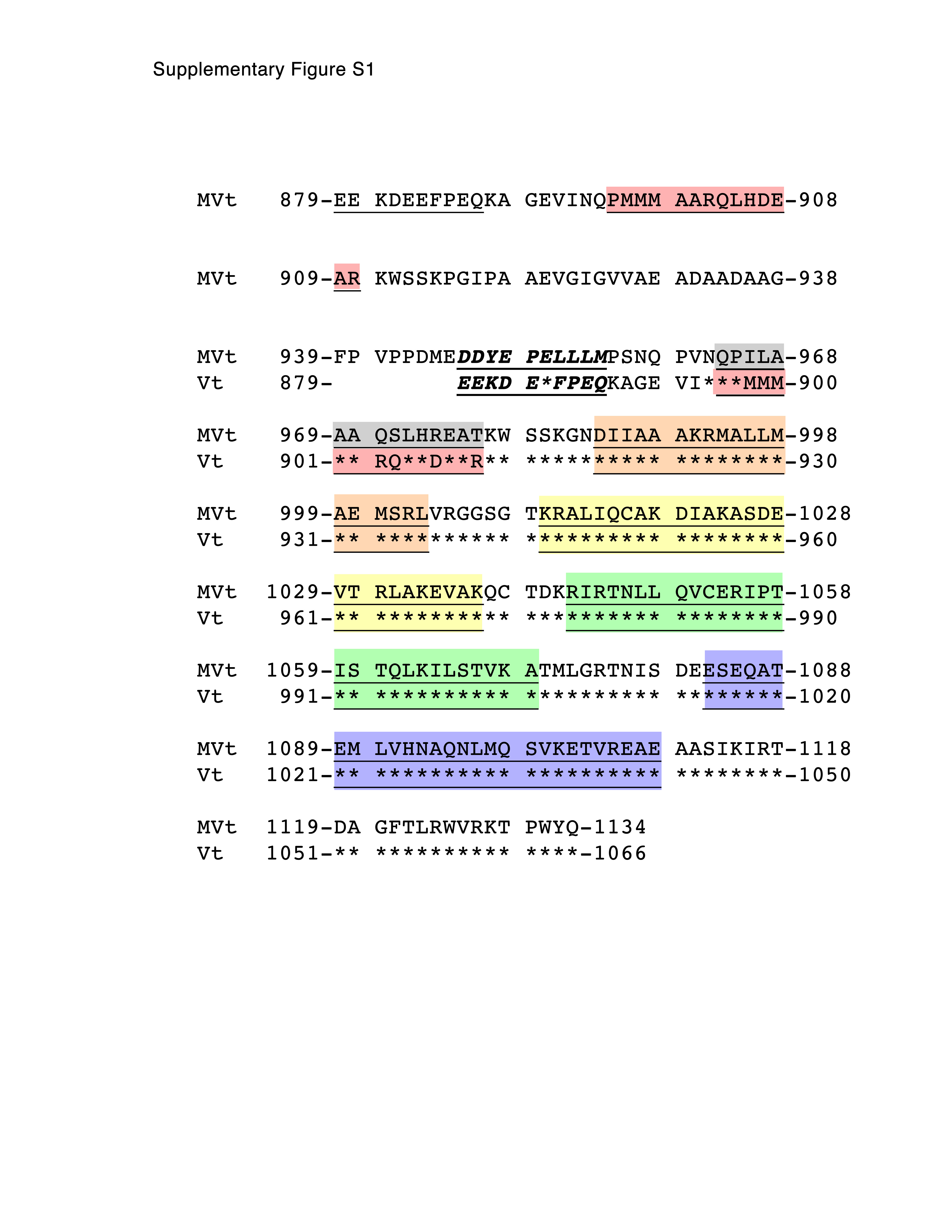

Supplement: Figure S1 — Helix replacement mechanism in metavinculin. Structure-based sequence alignment of the α-helices of vinculin (Vt) and metavinculin (MVt) tail domains aligns the Vt α-helix H1 with the MVt α-helix H1′ and their respective N-terminal extended coils. This was a surprising revelation from the crystal structure since it was assumed that the α-helix H1 that is identical in sequence in the two isoforms would also be structurally conserved. The α-helices of MVt are underlined and colored in grey (H1′), red (H1), orange (H2), yellow (H3), green (H4), and blue (H5). The structurally equivalent extended coil regions are in bold, underlined, and italicized. The Vt extended coil region (residues 879–888) is underlined in the MVt sequence. Identical residues in MVt and Vt are indicated by an asterisk in the Vt sequence. (3.44 MB TIF) [file pone.0010679.s002.tif]

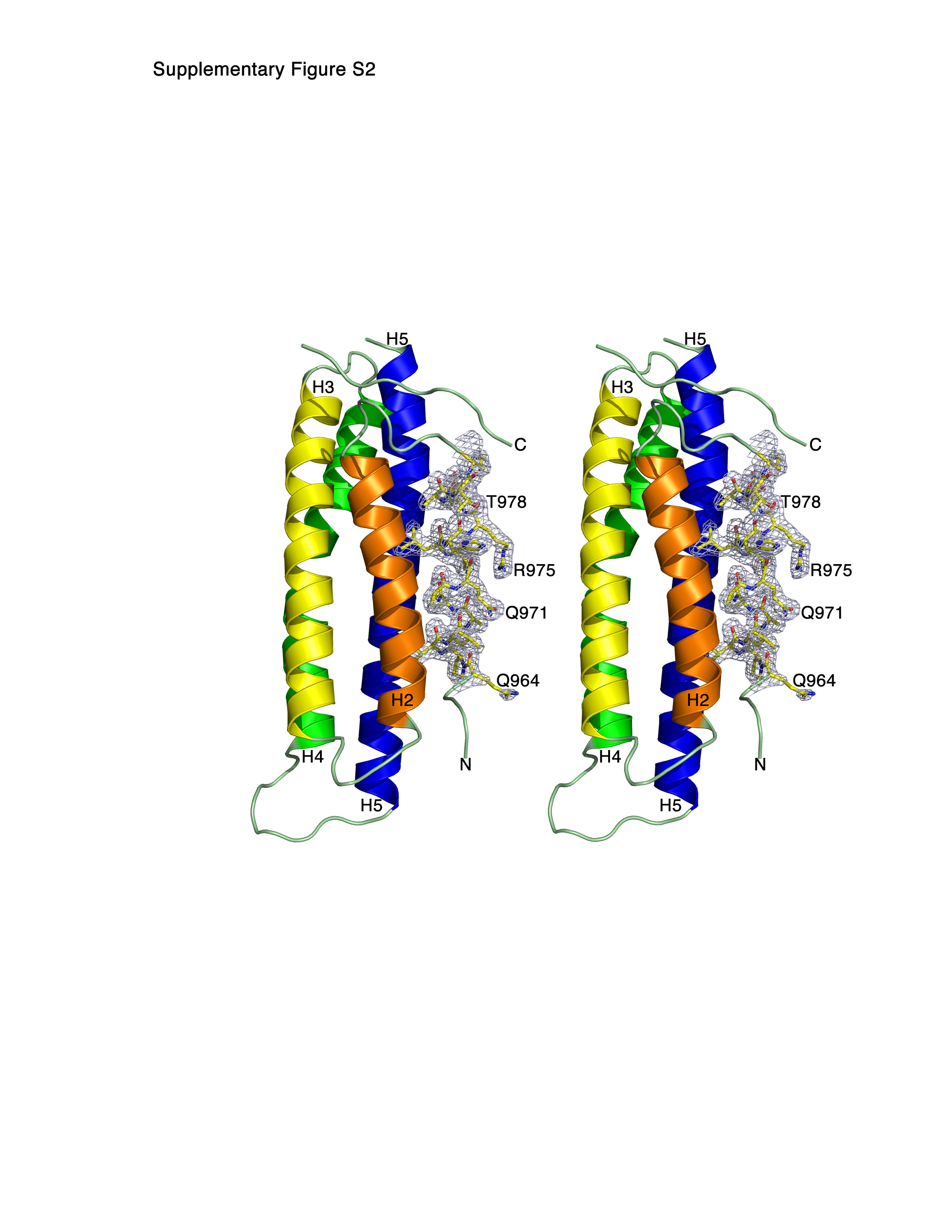

Supplement: Figure S2 — MVt electron density map. Stereo view of the final 2Fobs-Fcalc electron density map at 2.2 Å of the metavinculin tail domain contoured at 1σ around the replaced α-helix H1′ represented in sticks. The remaining four α-helices are shown as a cartoon. The α-helices of MVt are colored in orange (H2; residues 986–1,004), yellow (H3; residues 1,012–1,038), green (H4; residues 1,043–1,071), and blue (H5; residues 1,081–1,114) while the α-helix H1′ (residues 964–979) is shown in sticks. (2.52 MB TIF) [file pone.0010679.s003.tif]

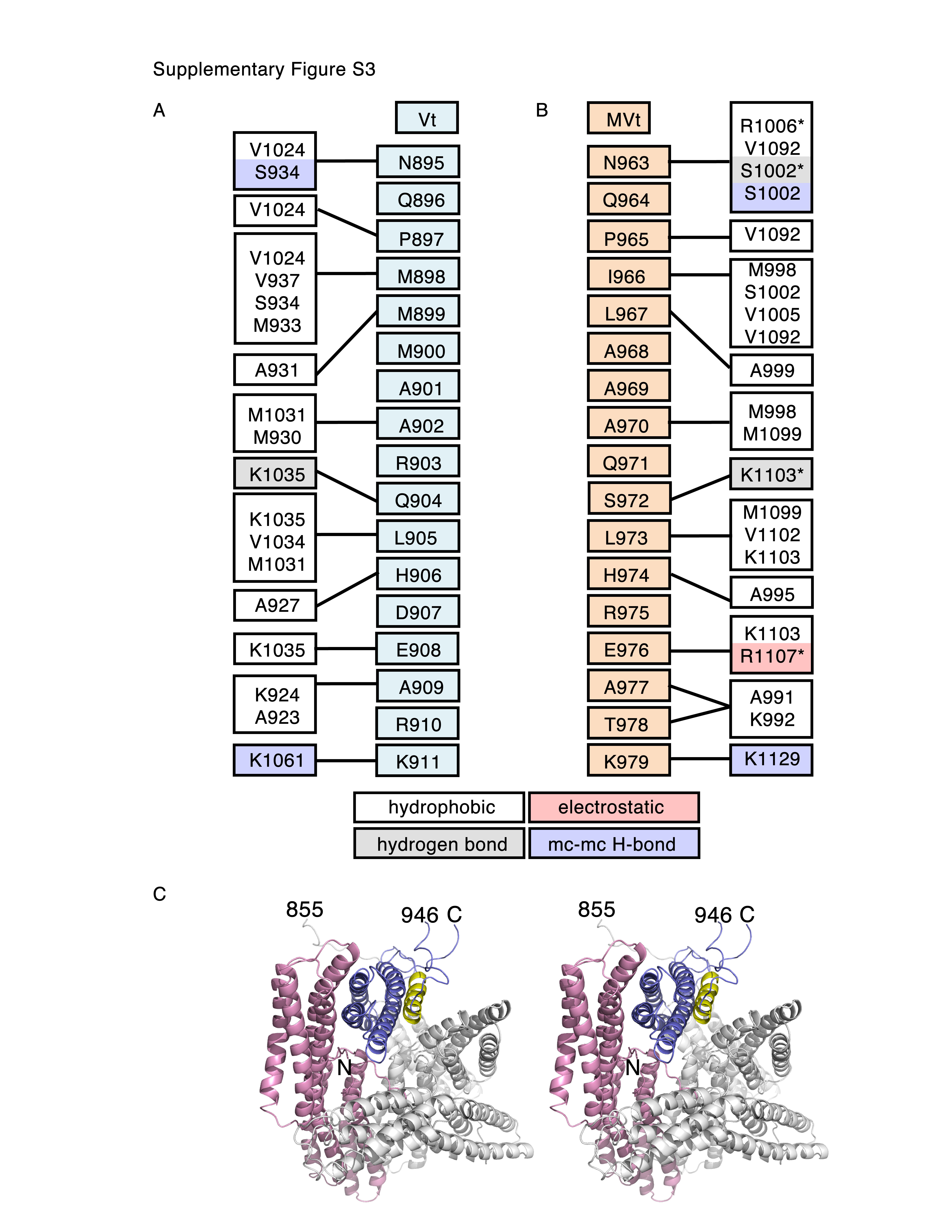

Supplement: Figure S3 — Intramolecular interactions of α-helices H1 in vinculin versus H1′ in metavinculin. Schematic of the intramolecular interactions of α-helix H1 of Vt (A) versus those of α-helix H1′ of MVt (B). The residues binding to Vt α-helix H1 (boxed in pale blue) or MVt α-helix H1′ (boxed in peach) are shown on the left (A) or on the right (B) of the respective helices. Residues are distinguished according to the type of their interaction (hydrophobic, white; hydrogen bonds, gray; backbone hydrogen bonds, blue; electrostatic interactions, pink). The asterisks indicate additional interactions found in MVt (Ser-1002, Arg-1006, Arg-1107) or altered interactions compared to Vt (Ser-972 with Lys-1103 in MVt versus Gln-904 with Lys-1035 in Vt). C: Cartoon stereo drawing of the full-length metavinculin crystal structure. The head domain, VH, is shown in pink (Vh1 sub-domain; residues 1–258) and gray (residues 259–840) and the tail domain, MVt, is shown in blue (residues 946–963 and 980–1,132) and yellow (α-Helix H1′; residues 964–979). α-Helix H1′ is shown in yellow and is not involved in the Vh1∶MVt interaction. The vinculin and metavinculin structure, including the distinct α-helices H1 and H1′, superimpose well (as shown in the superposition depicted in Figure 1) and, accordingly, α-helix H1 in vinculin is also not involved in the Vh1∶Vt interface. The entire head domain (residues 1–843) of metavinculin shows a root mean square deviation (r.m.s.d.) of 1.57 Å for 575 Cα atoms when compared with that of vinculin, while the MVt domain (residues 946–1,132) exhibits an r.m.s.d. of 0.8 Å for 163 Cα atoms. The termini as well as the disordered region are labeled (“N” and “C”, and “855” and “946”, respectively). (6.93 MB TIF) [file pone.0010679.s004.tif]

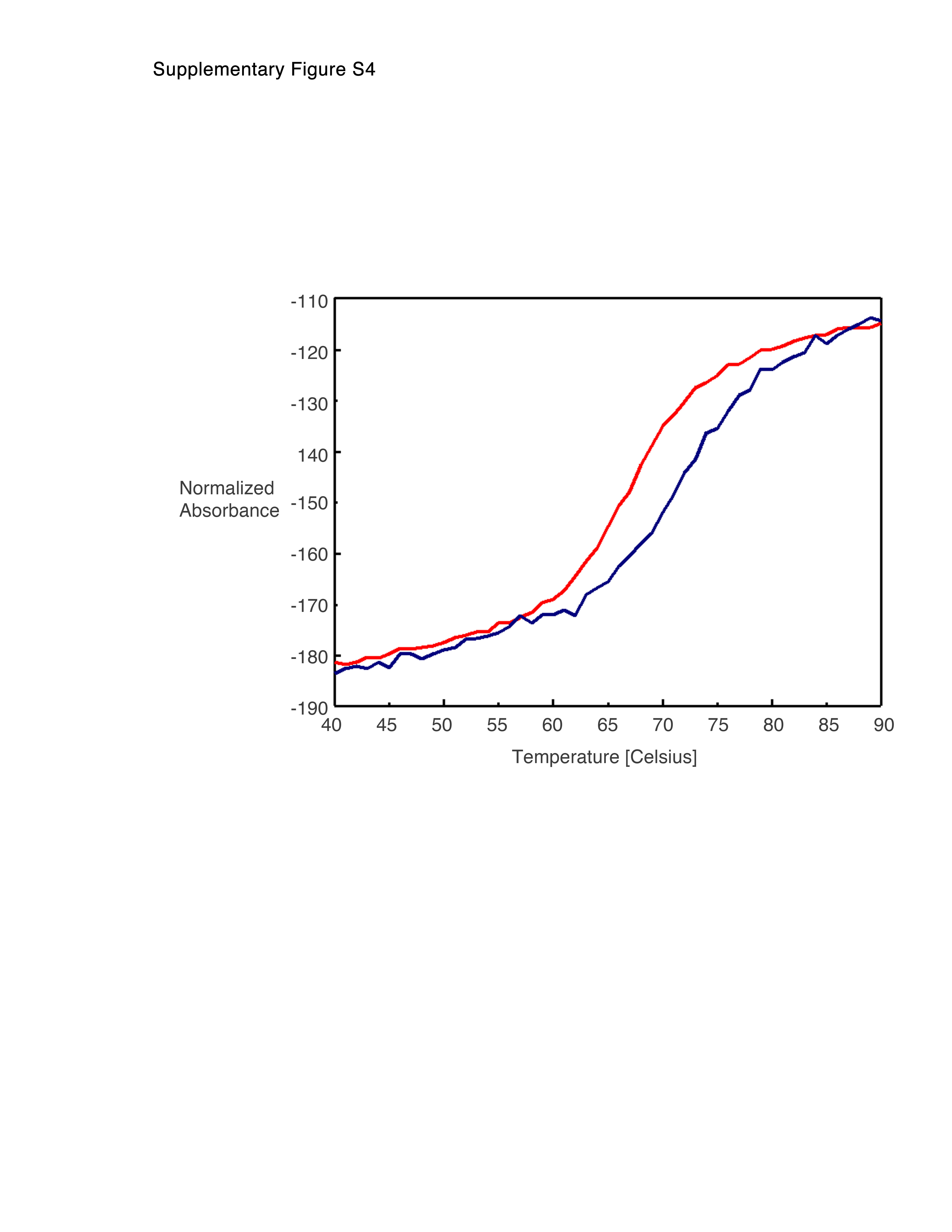

Supplement: Figure S4 — MVt is more stable than Vt. Normalized thermal unfolding curves of ΔN-Vt (residues 891–1066; red) and MVt-ΔH1 (residues 959–1130; blue) monitored at 222 nm are shown. The Tm values indicate that the metavinculin five-helix bundle (72°C) is significantly more stable compared to the vinculin five-helix bundle (67°C). (1.34 MB TIF) [file pone.0010679.s005.tif]

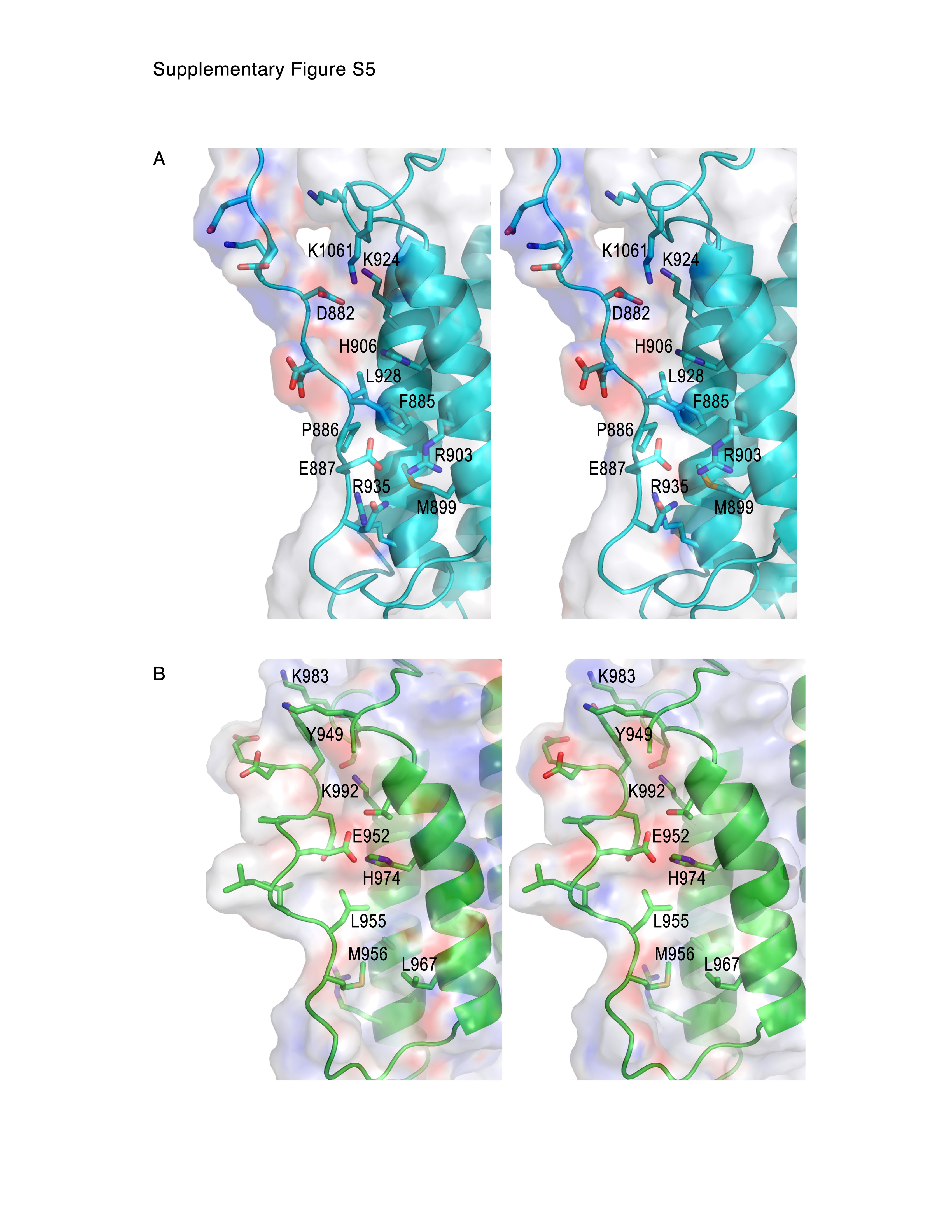

Supplement: Figure S5 — The extended coil in metavinculin engages in fewer interactions than that of the extended coil of vinculin. Stereo view of the Vt (A) and MVt (B) domains of the full-length metavinculin and vinculin crystal structures reveals that the extended coil in vinculin engages in more intradomain interactions compared to metavinculin. (6.92 MB TIF) [file pone.0010679.s006.tif]

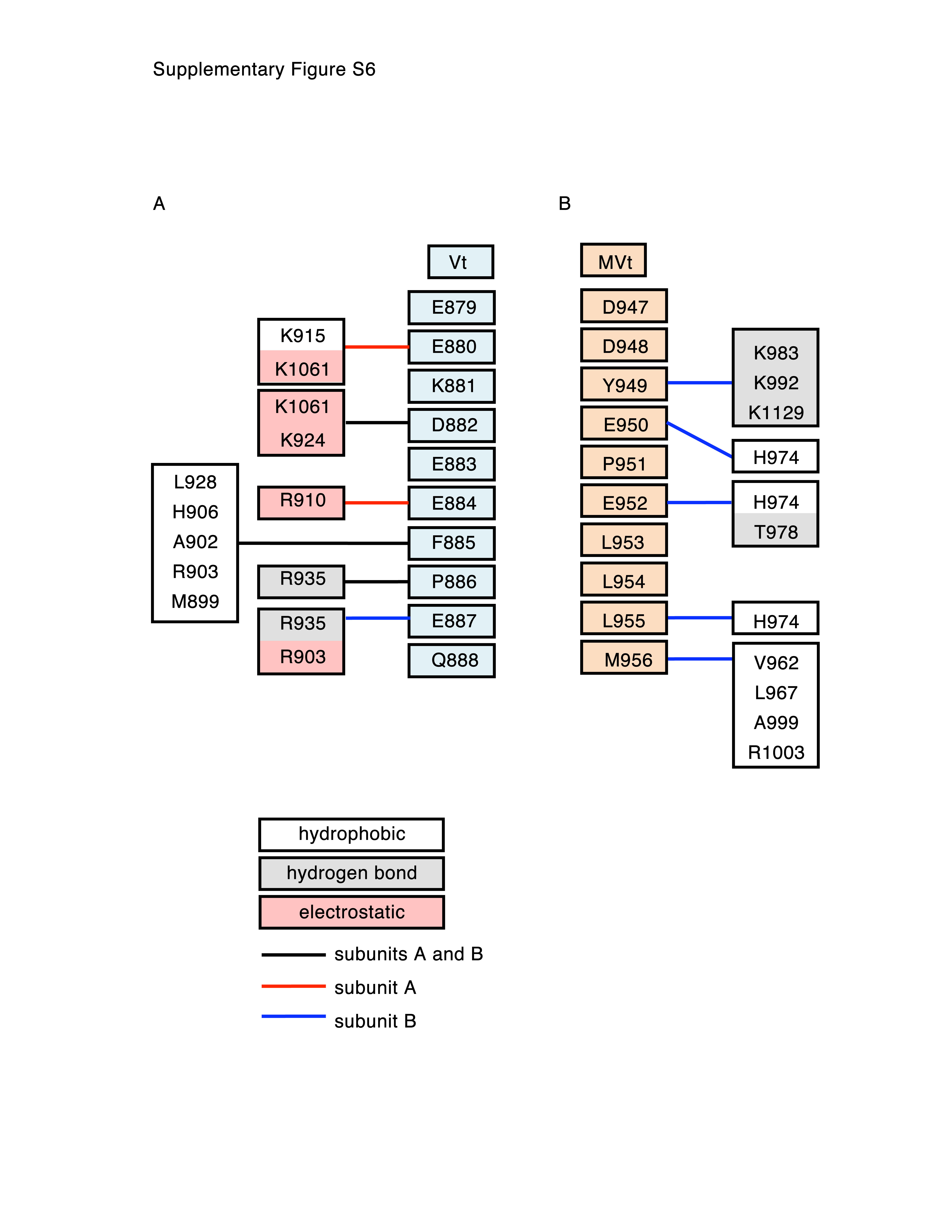

Supplement: Figure S6 — Fewer intramolcular interactions of the extended coil of metavinculin compared to the extended coil of vinculin. Schematic of the intramolecular interactions of the extended coil of vinculin (A) versus those of metavinculin (B). A: Six extended coil residues (three of these in only one subunit, indicated by the red and blue lines) interact with thirteen Vt residues, including five electrostatic interactions. B: Five metavinculin-specific extended coil residues engage in eleven hydrophobic/hydrogen bonding interactions. Indeed, these interactions are only seen in one subunit (indicated by the blue line), as the metavinculin-specific extended coil is disordered in the other subunit. (2.13 MB TIF) [file pone.0010679.s007.tif]

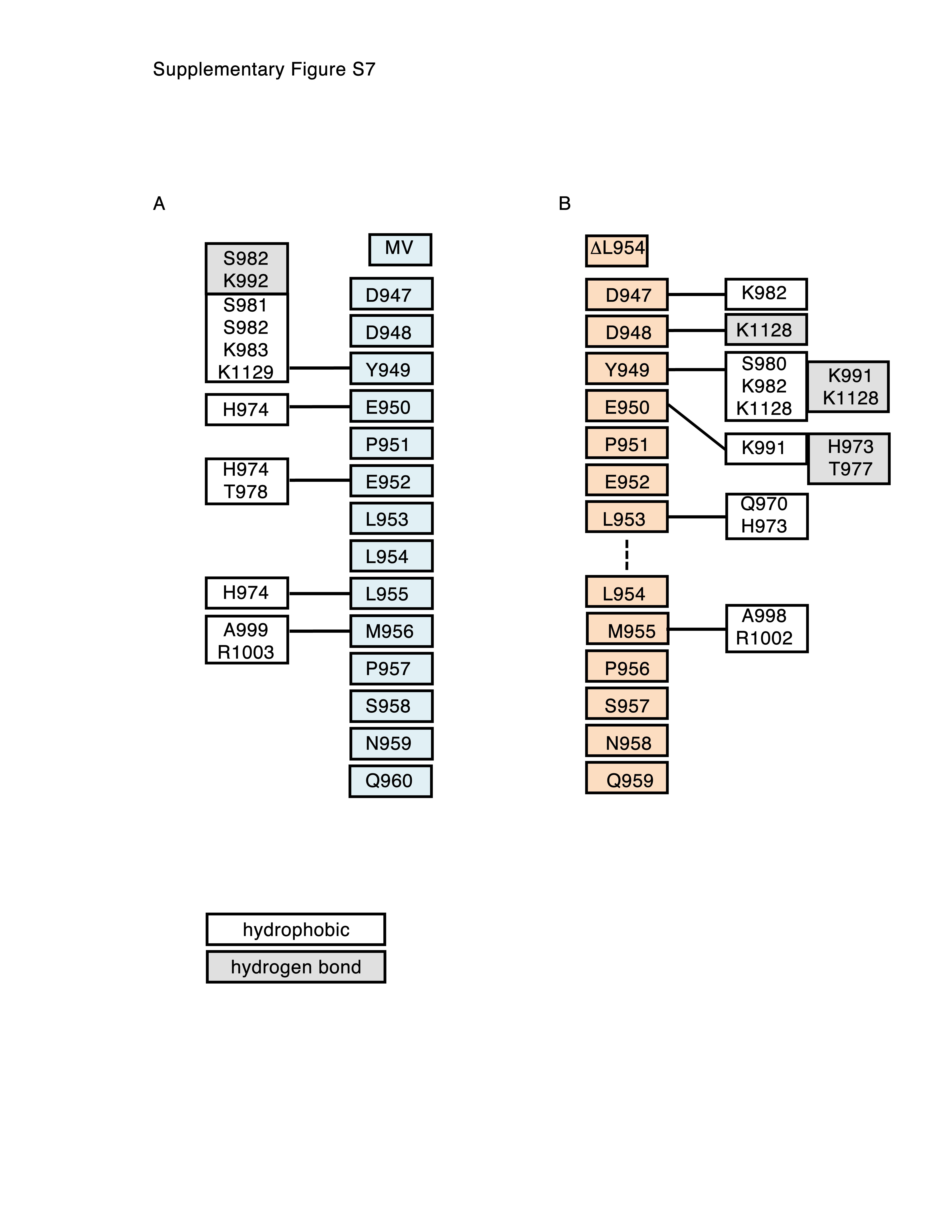

Supplement: Figure S7 — Intramolecular interactions of the extended coil in metavinculin and in the Leu954 deletion metavinculin mutant associated with cardiomyopathies. Residues binding to the wild type extended coil (A; boxed in light blue) or mutant metavinculin (B; boxed in peach) are shown on the left (A) or right (B) of the respective coils. Residues are distinguished according to the type of their interaction (hydrophobic, white; hydrogen bonds, gray). The deletion of Leu-954 is indicated by a dotted line in panel B. Due to the deletion, the numbering for residues 955–1,134 of wild type metavinculin in the ΔL954 mutant is 954–1,133. (2.17 MB TIF) [file pone.0010679.s008.tif]

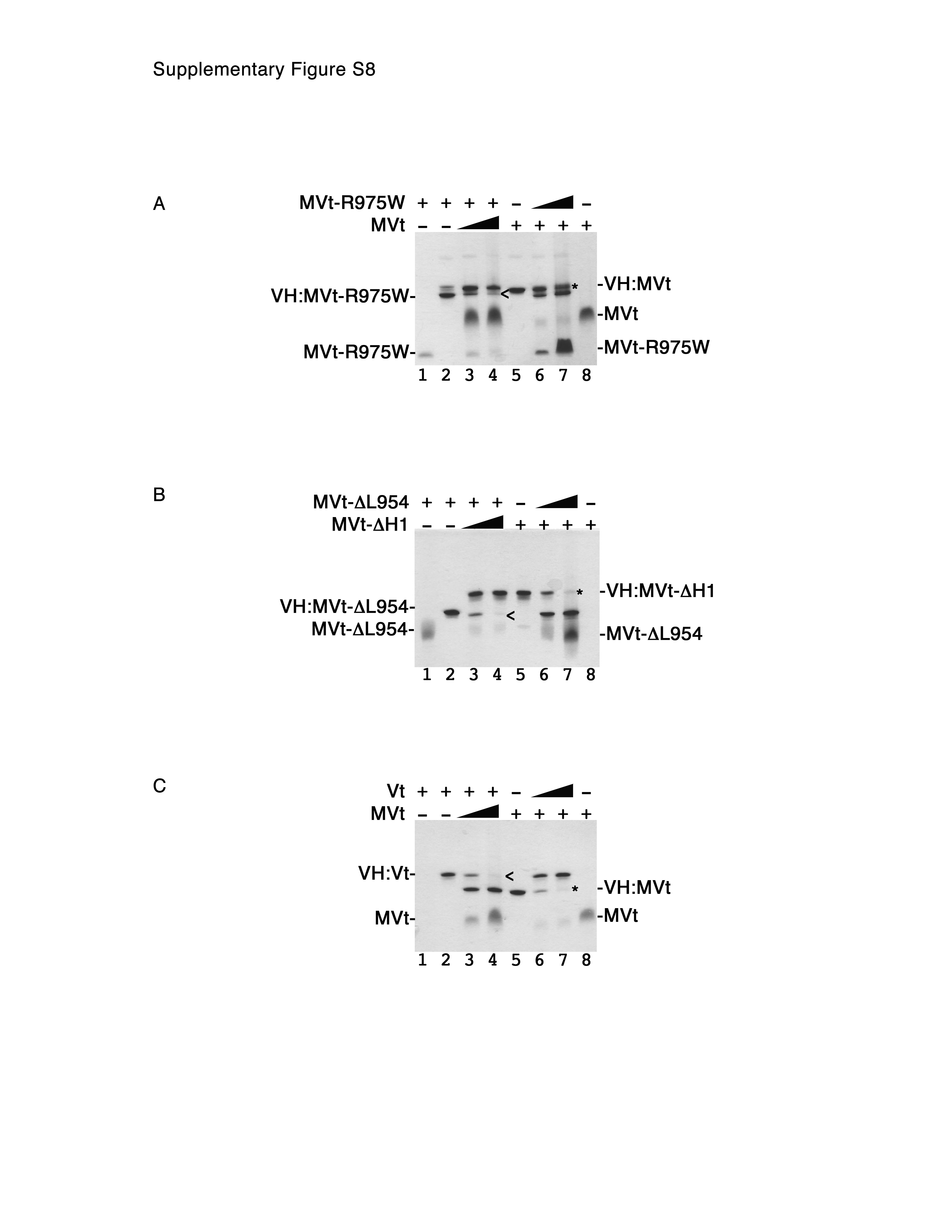

Supplement: Figure S8 — Helix replacement does not affect the head∶tail interaction. Reciprocal tail displacement native gel analyses of VH (residues 1–843) in complex with (A) MVt-Arg975Trp, (B) MVt-ΔLeu954, and (C) Vt versus MVt (A and C) or MVt-ΔH1(B). VH in complex with MVt or MVt-ΔLeu954 migrate similarly (ESR personal communication); thus, MVt-ΔH1 was used instead of MVt to be able to distinguish the complexes. Vt and MVt-ΔH1 do not migrate into the gel due to their basic pIs of 9.32 and 9.89, respectively. Competing tail domains were titrated (arrows) into preformed complexes at 2- and 10-fold molar excess. Notably, the DCM-associated metavinculin mutant MVt-R975W (A) shows the weakest head∶tail interaction while the tail domains of the two vinculin isoforms or the ΔLeu deletion mutant do not affect this interaction. Representative analyses of native gels are shown. The less-than sign (<) and asterisk highlight the displaced VH∶tail domain complexes. MVt displaces MVt-Arg975Trp more readily (A), MVt-ΔLeu954 and MVt-ΔH1 displace each other similarly (B), and MVt and Vt displace each other equally (C). (2.17 MB TIF) [file pone.0010679.s009.tif]

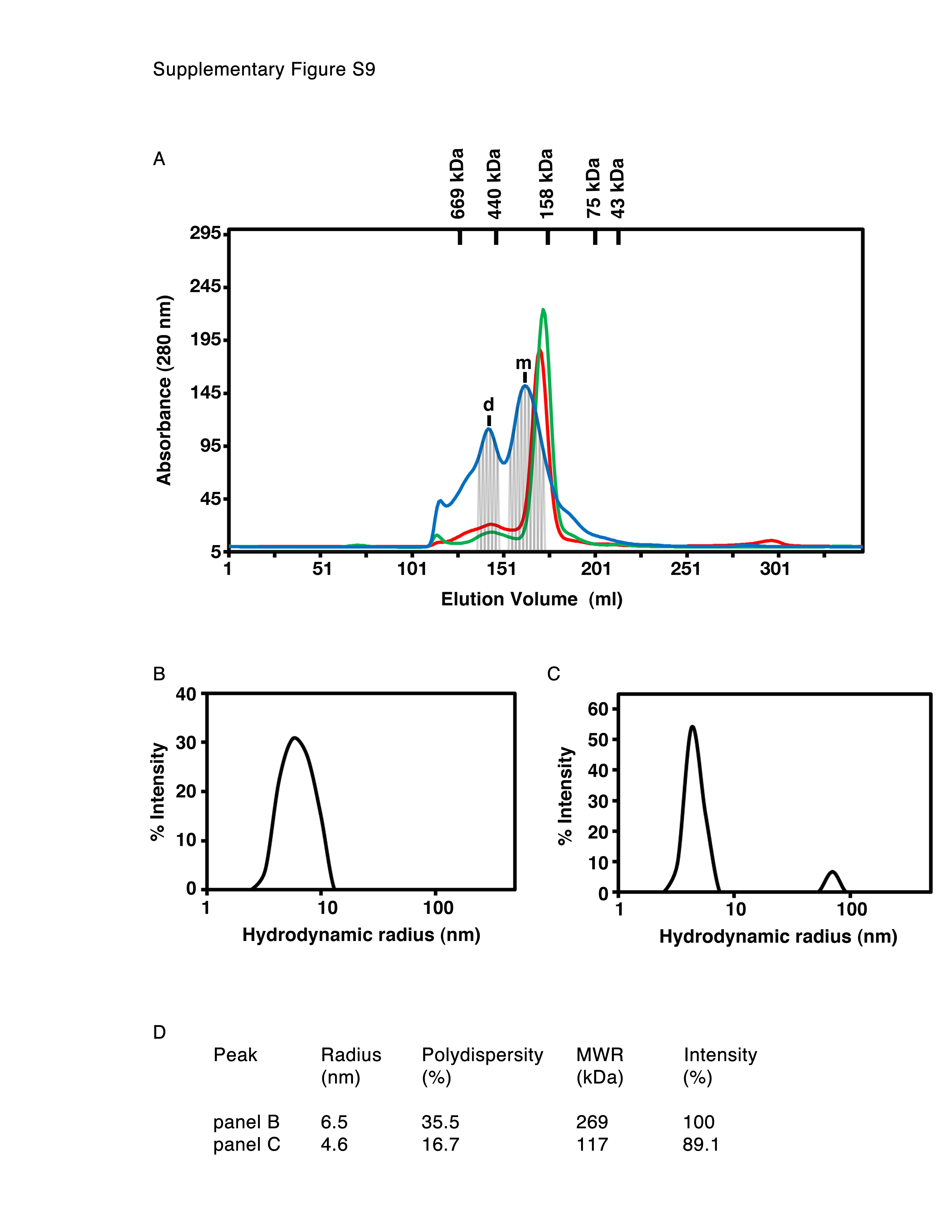

Supplement: Figure S9 — α-Helix H1′ impairs metavinculin oligomerization. A: Chromatogram of size exclusion chromatography of full length metavinculin (red; 123.8 kDa), MV-ΔH1 (green; 121.3 kDa), and MV-ΔH1′ (blue; 121 kDa) on a Hiload 26/60 Superdex 200 column calibrated with standard molecular weight markers (thyroglobulin, 669 kDa; ferritin, 440 kDa; aldolase, 158 kDa; conalbumin, 75 kDa; and ovalbumin, 43 kDa). The elution positions of the standard proteins are indicated on the chromatogram. The shaded areas for the dimer (‘d’) and monomer (‘m’) elutants of MV-ΔH1′ indicate the fractions pooled for dynamic light scattering (panels B and C) and native gel (Figure 4A) experiments. B: and C: Analysis of the oligomeric state of the two species, ‘d’ and ‘m’, of MV-ΔH1′ separated by gel filtration (A) by dynamic light scattering (DLS) at 23°C. DLS measurements were carried out at a scattering angle of 90° on a Dynapro Titan (Wyatt technologies) dynamic light scattering apparatus. D: The calculated molecular weight (MWR) for peak fractions of the two species of MV-ΔH1 separated by gel filtration (A) as determined by dynamic light scattering. Values provided correspond to major peaks as obtained by DLS experiment. The relative intensity in this panel represents the relative amount of light scattered by each population of species in solution while the relative intensities in panels B and C indicate the relative amount of light scattered by the bin compared to the other bin and thus differ in values for the same peak. (2.60 MB TIF) [file pone.0010679.s010.tif]
